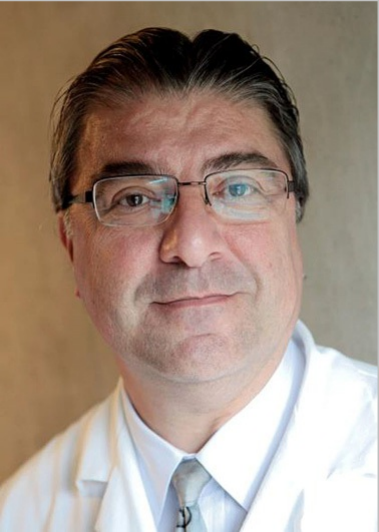# Organic, Organometallic and Bioorganic Catalysts for Electrochemical Reduction of CO_2_

**DOI:** 10.1002/cphc.201700148

**Published:** 2017-05-31

**Authors:** Dogukan Hazar Apaydin, Stefanie Schlager, Engelbert Portenkirchner, Niyazi Serdar Sariciftci

**Affiliations:** ^1^ Linz Institute for Organic Solar Cells (LIOS) Institute of Physical Chemistry Johannes Kepler University Linz A-4040 Linz Austria; ^2^ Institute of Physical Chemistry University of Innsbruck A-6020 Innsbruck Austria

**Keywords:** bioelectrocatalysis, CO_2_ reduction, electrocatalysis, heterogeneous catalysis, organic semiconductors

## Abstract

A broad review of homogeneous and heterogeneous catalytic approaches toward CO_2_ reduction using organic, organometallic, and bioorganic systems is provided. Electrochemical, bioelectrochemical and photoelectrochemical approaches are discussed in terms of their faradaic efficiencies, overpotentials and reaction mechanisms. Organometallic complexes as well as semiconductors and their homogeneous and heterogeneous catalytic activities are compared to enzymes. In both cases, their immobilization on electrodes is discussed and compared to homogeneous catalysts in solution.

## 1 Introduction

The increased presence of greenhouse gases in the atmosphere is a major problem that needs to be addressed for a sustainable future. Of the greenhouse gases, carbon dioxide is the principal concern due to its residence time in the atmosphere, which is estimated to be years, whereas water vapor has a residence time of days.^1^ Humans use carbon in a linear way, transforming fossil fuels into atmospheric CO_2_. Without additional support for natural photosynthetic systems to fix atmospheric CO_2_, within a couple of decades, human civilization will have returned to the atmosphere what natural photosynthesis had fixed over millions of years. As such, humans need a cyclic way of using CO_2_ to have sustainable future.

Two main approaches have been suggested for addressing this issue—carbon capture and sequestration (CCS)^2^ and carbon capture and utilization (CCU).^3^ CCS describes the capture of CO_2_ at its human‐made origin (e.g., factories and power plants) and its sequestration in underground (e.g., in oil wells and under ocean and underground bedrocks), without utilizing CO_2_ as such.^4^ This method is expensive and will not result in a cyclic use of carbon.

In contrast, the CCU approach covers a broad number of processes that can be applied to address the issue in which CO_2_ is not only captured but also used as a feedstock for various chemical products such as formic acid, carbon monoxide, methanol, and methane.^5^, ^6^, ^7^, ^8^, ^9^, ^10^ Using this method, a carbon‐neutral fuel cycle might be realized. For example, a synthetic fuel, which has been created by recycling the CO_2_ from the atmosphere with the help of renewable energies, would release exactly the same amount of carbon to the atmosphere as used in its manufacture in the first instance. Therefore, the recycling of CO_2_ into synthetic fuels is a carbon‐neutral energy vector provided that only renewable energies such as solar and wind power are used to input energy into the recycling process.

This Review summarizes the recent efforts to realize the photoelectro‐ and electrocatalytic conversion of CO_2_ into synthetic fuels using organometallic, organic and bioorganic catalysts. We intentionally excluded photocatalytic approaches, because they go beyond the space and scope of such a review. However, interested readers are advised to refer to Ref. 11 and related books and papers therein. This article is divided into two main chapters that describe the homogenous (catalyst and CO_2_ are in the same phase) and heterogeneous (the catalyst material is in the solid phase whereas CO_2_ is dissolved in the electrolyte solution) electrocatalytic, photoelectrocatalytic and bioelectrocatalytic methods toward converting CO_2_. In each chapter there are subsections summarizing what has been done in the field, including our contributions.

**Figure 1:**
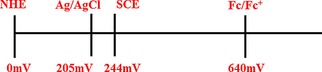
Reference electrode potentials versus the normal hydrogen electrode (NHE). Ag/AgCl electrode potential value is given for a 3 m KCl solution. The vacuum level for the determination of energy bands are set to −4.75 eV for NHE.^12^

Throughout this Review, the applied potentials and/or the potential ranges are reported versus reference electrodes, such as the normal hydrogen electrode (NHE), saturated calomel electrode (SCE), silver–silver chloride electrode (Ag/AgCl), and so forth, as they are reported in the original papers. For a comparison of these reference electrodes, readers can refer to the conversion bar shown in Figure 1.

Assessing the catalyst performance is of high importance for comparing different catalyst materials. There are several figures of merit given through this paper, namely, faradaic efficiency, catalytic rate constant, overpotential, and turnover number. Faradaic efficiency defines the selectivity of a catalyst towards a particular product and can be calculated as (moles of product/moles of electrons passed) × (number of electrons needed for conversion).

The catalytic rate constant *k* for a typical reaction of the following type [Eq. (1)]:

(1) $$n{}_{A}A\;+\;n{}_{B}B+...\;\to \;\mathrm{Products}$$

can be defined as rate=*k*[A]^*a*^[B]^*b*^, where *a* and *b* are usually (but not always) integers that are independent of the coefficients *n_A_* and *n_B_*. The dimensions of *k* depend on the exponential terms in the rate law. If we define the sum of the exponential terms of the concentration as *p* in the rate law (p=*a*+*b*+…) then *k* will have the dimensions of concentration^1−*p*^ per unit time.

Overpotential=applied potential−thermodynamic (or formal) potential for conversion.

Turnover number (TON)=moles of desired product/number of catalytically active sites (or moles of catalyst).

In this Review, we aim to give a broad overview of the field for researchers who have been working on the topic for many years as well as researchers who are starting out and would like to pursue this type of research.

## 2 Homogeneous Electrocatalysis for CO_2_ Reduction

This section of the Review covers the use of different catalysts—organometallic complexes, purely organic compounds, or bioactive materials—that are used electrochemically/photoelectrochemically. These catalysts are used homogeneously, which means they are in the same phase as the CO_2_ to be reduced.

### 2.1 Rhenium‐ and Manganese‐Containing Organometallic Complexes

Organometallic complexes are one of the most popular classes of materials in the field of CO_2_ reduction. Although there are many reported examples having varied molecular structure, polypyridine ligands are extensively used by many scientists in the field. Covering all of the reported polypyridine complexes would exceed the space and scope of this Review, therefore we focus on rhenium‐ and manganese‐containing complexes. However, we encourage interested readers to refer to one of the latest review articles summarizing the polypyridine ligands used for CO_2_ reduction.^13^ Among the polypyridine complexes, Re‐containing complexes are of wide interest.

**Figure 2:**
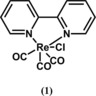
The chemical structure of Lehn's catalyst, [Re(bpy)(CO)_3_Cl].

The first of the Re‐containing complexes was reported by Hawecker, Lehn and Ziessel in 1984.^14^ In their paper, the authors describe their findings on [Re(bpy)(CO)_3_Cl] (bpy=2,2′‐bipyridine), which had been introduced as a homogeneous photocatalyst by the same group previously^15^ for the electrochemical reduction of carbon dioxide to carbon monoxide. Hawecker et al. showed that [Re(bpy)(CO)_3_Cl] (Figure 2) can produce 32 mL of CO if held at a potential of −1250 mV (vs. NHE) for 14 h without degradation, giving a remarkable faradaic efficiency of 98 % and a TON of 300. The authors note that the complex gives the highest efficiency if a mixture of DMF/H_2_O (9:1) is used together with 0.1 m Et_4_NCl as the supporting electrolyte. If no water was added, CO production was observed to be much slower, leveling off after a few hours.^14^ This study was a milestone in the field of carbon dioxide reduction and inspired many subsequent investigations.

Although the study of Hawecker and co‐workers set a milestone in the field, in the initial paper, the mechanism behind the process was not elaborated in detail. However, the study did have an important comparative experimental aspect in which electrolyte solutions with and without water were used. This was an important hint for the subsequent studies. Sullivan et al. performed a detailed study on the [Re(bpy)(CO)_3_Cl] complex to clarify the mechanism.^16^ Their report describes the electrochemical behavior of the complex as well as the related derivatives, which led the authors toward two independent pathways for the electrochemical reduction of carbon dioxide. The derivatives used in the study were represented with the general formula [Re(bpy)(CO)_3_L]^*n*+^, where L=4‐ethylpyridine (*n*=1), Cl^−^ (*n*=0) or hydride (*n*=0). The authors showed that the variation of the ligand did not affect the redox potential of the first reversible reduction peak, which was observed at approximately −1120 mV (vs. NHE), and concluded that this peak originates from the reduction of bipyridine (bpy). The second (irreversible) reduction peak potential varied with the changing ligands L suggesting that this process is metal based. Coulometry and bulk electrolytic reduction at −1100 to −1200 mV (vs. NHE) demonstrated that the first reduction is a one‐electron process. However, this process is coupled to the formation of the sparingly soluble green dimer [Re(bpy)(CO)_3_]_2_. The authors characterized this dimer by UV/Vis, IR and ^1^H NMR spectroscopy as well as elemental analysis. They also produced this dimer by chemical synthesis to further support their spectroscopic conclusions. The mechanism for the formation of the Re–Re dimer proposed by Sullivan et al.^16^ can be summarized as follows [Eqs. (2), (3), (4), (5)]:

(2) [Re(bpy)(CO)3Cl]+e-←→[Re(bpy•-)(CO)3Cl]

(3) [Re(bpy•-)(CO)3Cl]→fast[Re0(bpy)(CO)3Cl]-

(4) [Re0(bpy)(CO)3Cl]-→slow[Re(bpy)(CO)3]+Cl-

(5) 2[Re(bpy)(CO)3]→[Re(bpy)(CO)3]2

**Figure 3:**
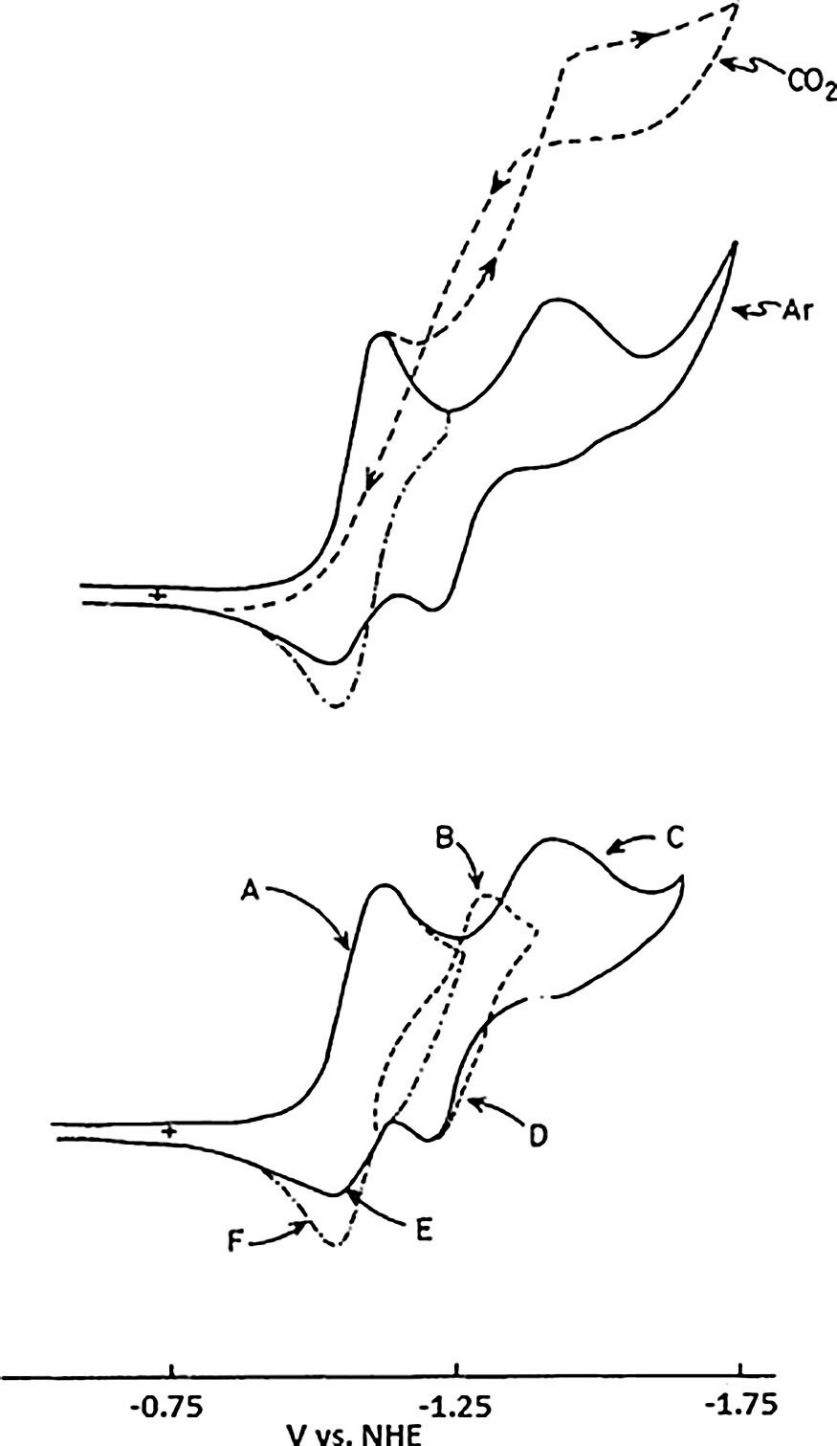
Top: Cyclic voltammograms of Re complexes under argon and under CO_2_. Bottom: Different electrochemical processes of Re complex measured in 0.1 m Tetrabutylammonium hexafluorophosphate (TBAH) in MeCN with a scan rate of 200 mV s^−1^ Reproduced with permission from Ref. 15.

The lack of redox activity, indicating the formation of dimer species in the cyclic voltammogram of the complex, was explained by the slow rate of the process. The authors suggest that loss of Cl^−^ might be preceded by intramolecular electron transfer to a metal‐based dσ* orbital [Eq. (3)], which, as a result, might facilitate metal–metal bond formation. The authors conducted a constant‐potential experiment at −1500 mV (vs. NHE), which consumed two electrons per Re atom to give a red‐purple solution. This is believed to be the anionic form ([Re(bpy)(CO)_3_]^−^) of the complex. If CO_2_ was introduced into the electrolyte the first reversible peak at −1120 mV did not show a catalytic enhancement, suggesting that bpy does not take part in the catalytic reduction of carbon dioxide if a current enhancement in the second reduction peak of the complex is observed (Figure 3).

**Scheme 1:**
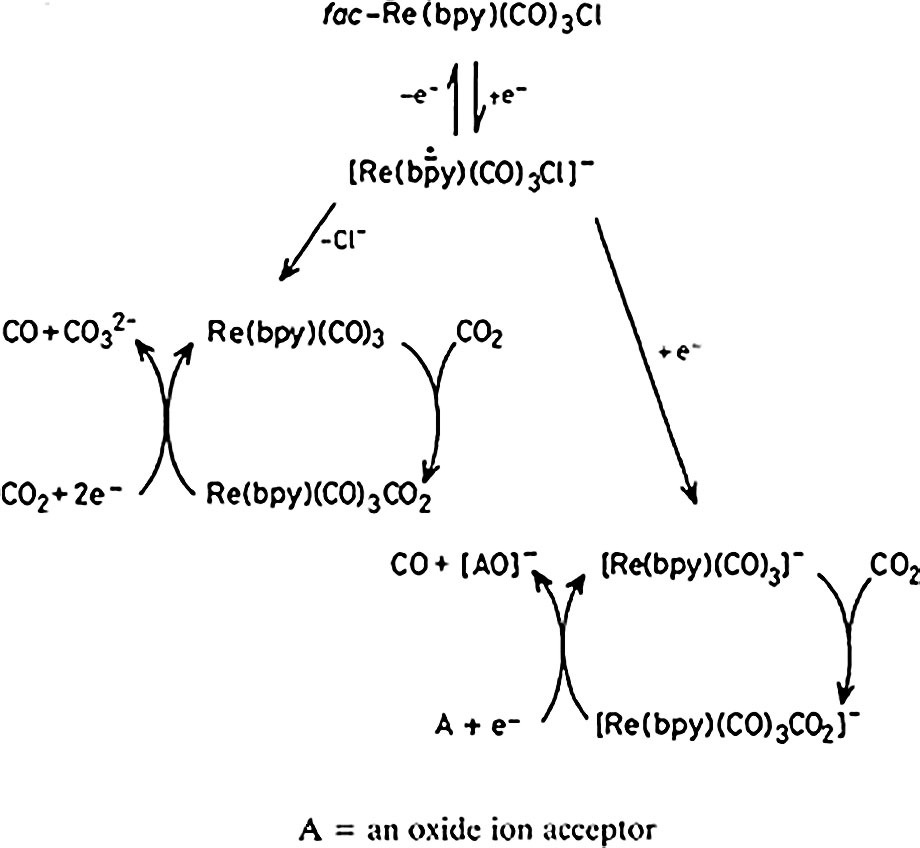
Reaction pathways suggested by Sullivan and co‐workers.^16^ Reproduced with permission from Ref. 15.

The authors summarize their findings in a two‐way reaction scheme (Scheme 1) and state that the first path starts with a radical form of [Re(bpy)(CO)_3_] or its solvated form [Re(bpy)(CO)_3_]**⋅**MeCN. They also conclude that dimer formation occurs only if CO_2_ is not present in the reaction medium, suggesting that carbon dioxide intercepts dimer formation. The second path (a two‐electron pathway) involves the anion [Re(bpy)(CO)_3_]^−^ and results in the production of CO with high current efficiency.

**Scheme 2:**
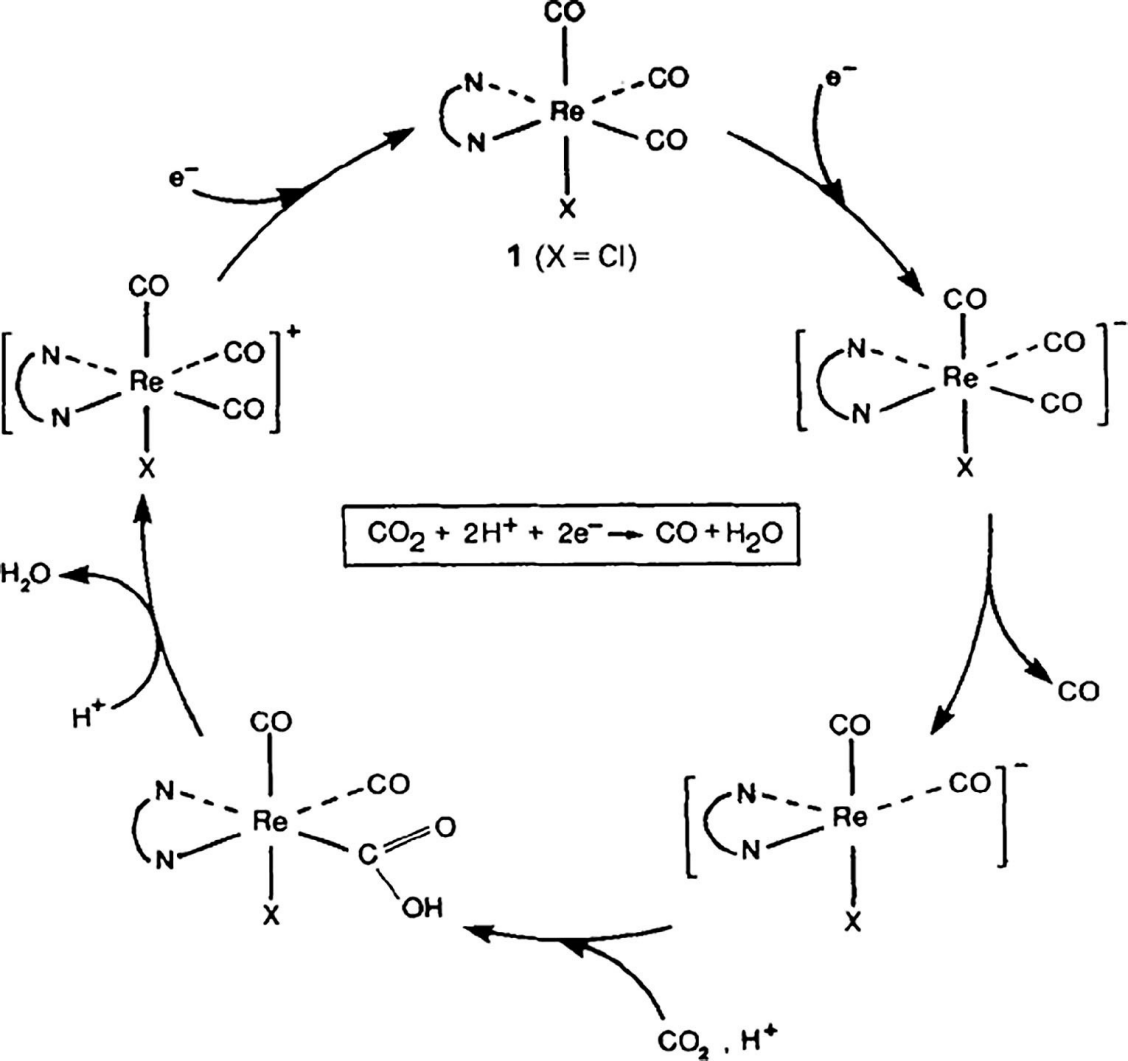
Reaction mechanism suggested by Hawecker, Lehn and Ziessel. Reproduced with permission from Ref. 16.

In 1986, Hawecker, Lehn and Ziessel published results that included the extended studies on mechanism and the origin of formed products.^17^ The authors argued that irradiation of the complex under optimum conditions led to a rhenium‐to‐bipyridine charge‐transfer excited state, which then was reductively quenched by a tertiary amine to give [Re(bpy)(CO)_3_Cl]^−^. They proposed that the same should be possible in an electrochemical system in which the Re complex is reduced at an electrode. They also showed that the maximum efficiency was reached if the reaction medium contained 10 % H_2_O. A water content exceeding 10 % caused a decrease in the efficiency, and formation of a green precipitate was observed. Their results are in agreement with those presented by Sullivan et al.,^16^ and they identified the green precipitate as the dimer of the complex. The authors also concluded their studies by suggesting a reaction mechanism for the formation of CO in the presence of water (Scheme 2).^17^

These studies shed light onto the possible mechanistic pathways leading to electrochemical reduction of carbon dioxide to carbon monoxide. However, there was still a debate on whether a 1 e^−^ or 2 e^−^ reduction pathway is preferred or if there are circumstances under which one of those two is preferred. Spectroelectrochemical IR studies^18^, ^19^, ^20^, ^21^ helped to clarify the mechanism further. Johnson and co‐workers studied the catalytic activity of both the radical [Re(bpy)(CO)_3_Cl]^.^ and the anion [Re(bpy)(CO)_3_]^−^ separately. Using ligands other than halides, they eliminated the potential problem of both mechanisms operating simultaneously due to the solvent effect. The radical [Re(bpy)(CO)_3_Cl]^.−^ could be stabilized at lower temperatures,^22^ however, at room temperature it transforms into [Re(bpy)(CO)_3_]^.^ due to loss of chloride. In the absence of strongly coordinating solvents or CO_2_ this species dimerizes into [Re(bpy)(CO)_3_]_2_, as confirmed by Johnson and co‐workers.^21^ In contrast to a previous study by Sullivan et al.^16^, Johnson and co‐workers discovered that the radical [Re(bpy)(CO)_3_(CH_3_CN)]^.^ is stable in acetonitrile and is involved in a two‐electron pathway for the electrochemical reduction of CO_2_. The authors provide spectroscopic evidence showing for the first time that CO_2_ does not interact directly with the radical anion [Re(bpy)(CO)_3_Cl]^.−^, which can only be regarded as the catalyst precursor, thus ruling out the one‐electron pathway. Nonetheless, a one‐electron mechanism cannot always be excluded. This pathway prevails in solvents such as THF or DMF, which cannot stabilize 18‐electron radicals [Re(bpy)(CO)_3_(solvent)]^.^ due to their weaker coordination abilities compared to acetonitrile. This might therefore subsequently lead to dimer formation.

**Figure 4:**
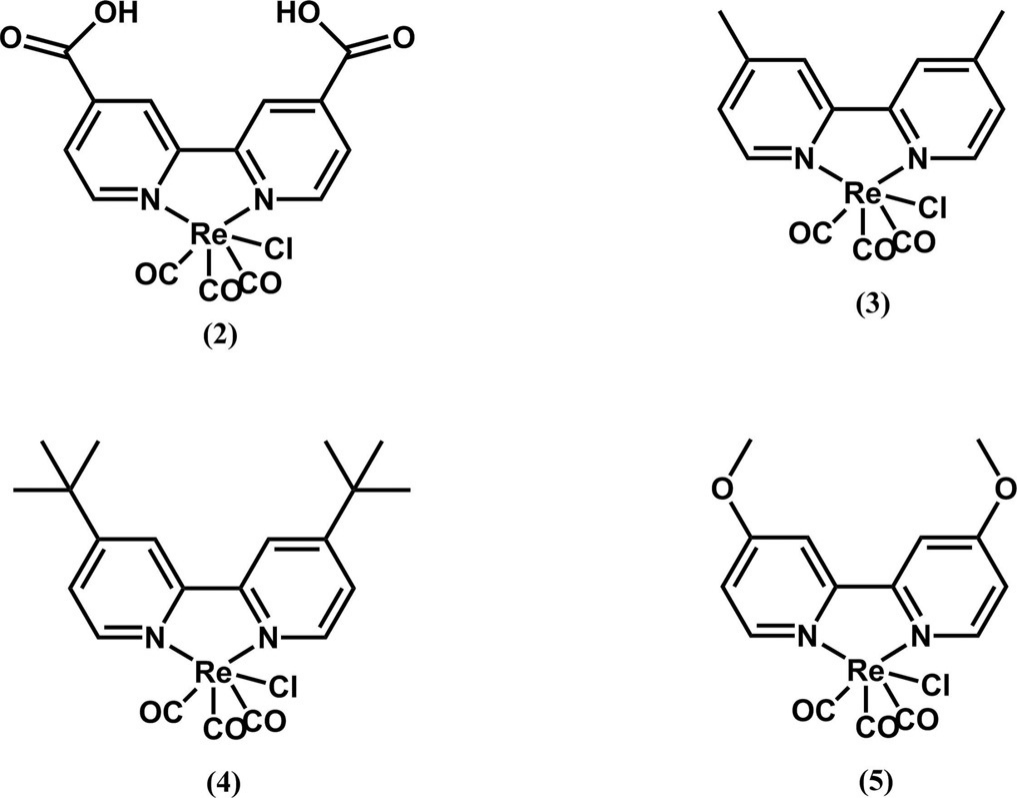
Chemical structures of various rhenium complexes investigated by Smieja and Kubiak.^23^

Later efforts have involved modification of the chemical structure of [Re(bpy)(CO)_3_Cl] in order to improve the catalytic activity. In 2010, Smieja and Kubiak reported their results for five different Re complexes, namely, [Re(bpy‐COOH)(CO)_3_Cl] (**1**), [Re(bpy)(CO)_3_Cl] (**2**), [Re(dmb)(CO)_3_Cl] (**3**, where dmb=4,4’‐dimethyl‐2,2’‐bipyridine), [Re(bpy‐*t*Bu)(CO)_3_Cl] (**4**), and [Re(bpy‐OMe)(CO)_3_Cl] (**5**), in terms of their catalytic activity and IR spectroelectrochemical analysis.^23^ The chemical structures of these Re complexes are shown in Figure 4.

The effect of the ligand on reduction of the complexes varied from −696 to −1241 mV (vs. NHE) for the first reduction and from −1481 to −1616 mV (vs. NHE) for the second reduction peak in complexes **1**–**5**. This difference in reduction peak potentials arises from the substituents in the 4 and 4′ positions having diverse electron‐donating/withdrawing abilities. The authors argued that the p*K*_a_ values of the parent pyridines determine the reduction potential and they reported the p*K*_a_ values as an increasing trend with electron‐donor character (4‐carboxypyridine, p*K*_a_=3.10; pyridine, p*K*_a_=5.17; 4‐methylpyridine, p*K*_a_=5.94; 4‐*tert*‐butylpyridine, p*K*_a_=5.99; 4‐methoxypyridine, p*K*_a_=6.62).^24^ If the solution was saturated with CO_2_ the complexes showed different increases in catalysis in their second reduction potential. Whereas complex **2** showed little to no current enhancement, **1** showed a 3.4‐fold increase. The authors compared **1** and **4**, as these complexes outperformed the others, and observed that compound **4** reached an 18.4‐fold current increase, surpassing the activity of **1** (Lehn's catalyst) by 3.5 times. The report also argued that catalytic activity towards CO_2_ reduction not only depends on the electrocatalyst reduction potential but also on many others. Although complex **5** has the most‐negative reduction potential, suggesting that it should possess a more nucleophilic Re center, it showed no catalytic activity towards CO_2_. This, according to Kubiak and co‐workers, suggests a possible π‐donor effect over a σ‐donor effect. Finally, the authors explained the high and longer electrocatalytic activity of **4** over **1** by the tendency of **4** to form a stable Re^0^ radical and undergo less dimerization compared to **1**; they supported this with IR spectroscopy data.

**Figure 5:**
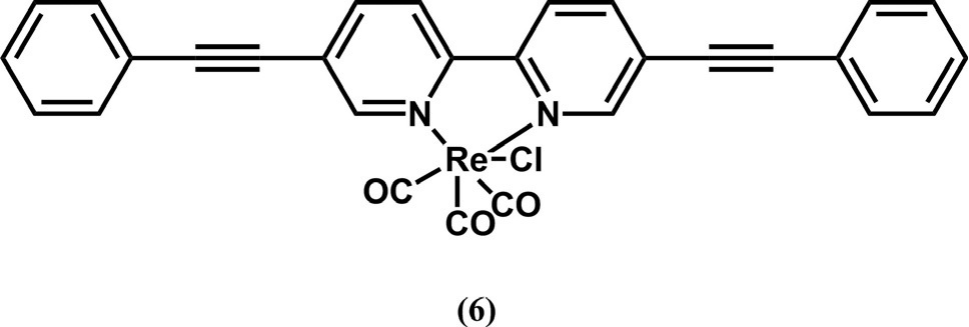
Chemical structure of [{5,5′‐bis(phenylethynyl)‐2,2′‐bipyridyl}Re(CO)_3_Cl] (**6**).

In 2012, Portenkirchner et al. investigated^25^ the effect of substituents at the 5 and 5′ positions (compound **6**, Figure 5) in a comparison to the work by Kubiak and co‐workers.^23^ They investigated the effect of extending the π‐conjugation on catalytic activity. The effect of extended conjugation on photophysical properties as well as the synthesis of **6** is published elsewhere.^26^

**Figure 6:**
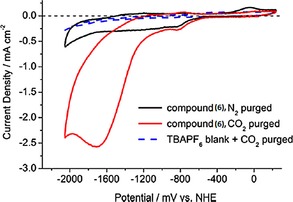
Electrochemical behavior of **6** under N_2_ and under CO_2_.^25^ Reproduced with permission from Ref. 24.

The electrochemical characteristics of **6** were investigated with cyclic voltammetry and the results are shown in Figure 6. Compound **6** did not show a clear peak separation between the first and the second reduction peak. However, it yielded a more positive reduction wave at around −750 mV (vs. NHE), which is 330 mV more positive compared to the first reduction peak of Lehn's catalyst (**1**). This might be explained by the increased π‐conjugation. If CO_2_ was introduced to a solution of **1**, the cyclic voltammogram showed a 4.5‐fold increase of the second reduction peak at −1750 mV (vs. NHE), whereas for **6**, a 6.5‐fold increase at the same potential was observed. Absolute current density was higher for **1** with a value of −3.47 mA cm^−2^, whereas it was −2.56 mA cm^−2^ for **6**. The authors also compared the catalytic rate constant *k* for these compounds. Lehn's catalyst **1** showed a catalytic rate constant *k* of 60 m ^−1^ s^−1^, whereas compound **6** yielded a *k* value of 220 m ^−1^ s^−1^. Finally, the authors compared the electrocatalytic and photocatalytic performance of the two compounds. Experiments were conducted in electrochemical cells having an H geometry. The amount of CO, as the expected product, was quantified by gas chromatography and FTIR transmission techniques. The amount of dissolved CO in the electrolyte solution was also estimated using Henry's law with a Henry constant *k*_H_ of 2507 atm mol^−1^ solvent mol^−1^ CO.^27^ Compound **6** showed a faradaic efficiency of 45 %, whereas Lehn's catalyst showed an efficiency of 50 % if the potential was held constant at −1950 mV (vs. NHE).^25^

In a further study, Portenkirchner et al. compared different molecular structures having Re as the metal center for electrocatalytic CO_2_ reduction as well as photocatalytic reduction.^28^ They used Lehn's catalyst **1** as the benchmark compound, adopted the 4,4′‐dicarboxy‐substituted bpy from the study of Kubiak et al.,^23^ and introduced the compound [{5,5′‐bis[(2,6‐bis‐octyloxy‐4‐formyl)phenylethinyl]‐2,2′‐bipyridyl}Re(CO)_3_Cl]. The authors counter argue the previous study^23^ by showing that the carboxy‐substituted complex can display a catalytic current enhancement if CO_2_ is introduced into the medium. However, to the authors’ surprise the compound did not yield products and lost its catalytic activity within minutes upon applying a bias; the authors attributed this to instability of the compound. [{5,5′‐Bis[(2,6‐bis‐octyloxy‐4‐formyl)phenylethinyl]‐2,2′‐bipyridyl}Re(CO)_3_Cl] did not show a significant current enhancement upon contact with CO_2_. However, no explanation was given for the lack of catalytic activity of this molecule.

**Figure 7:**
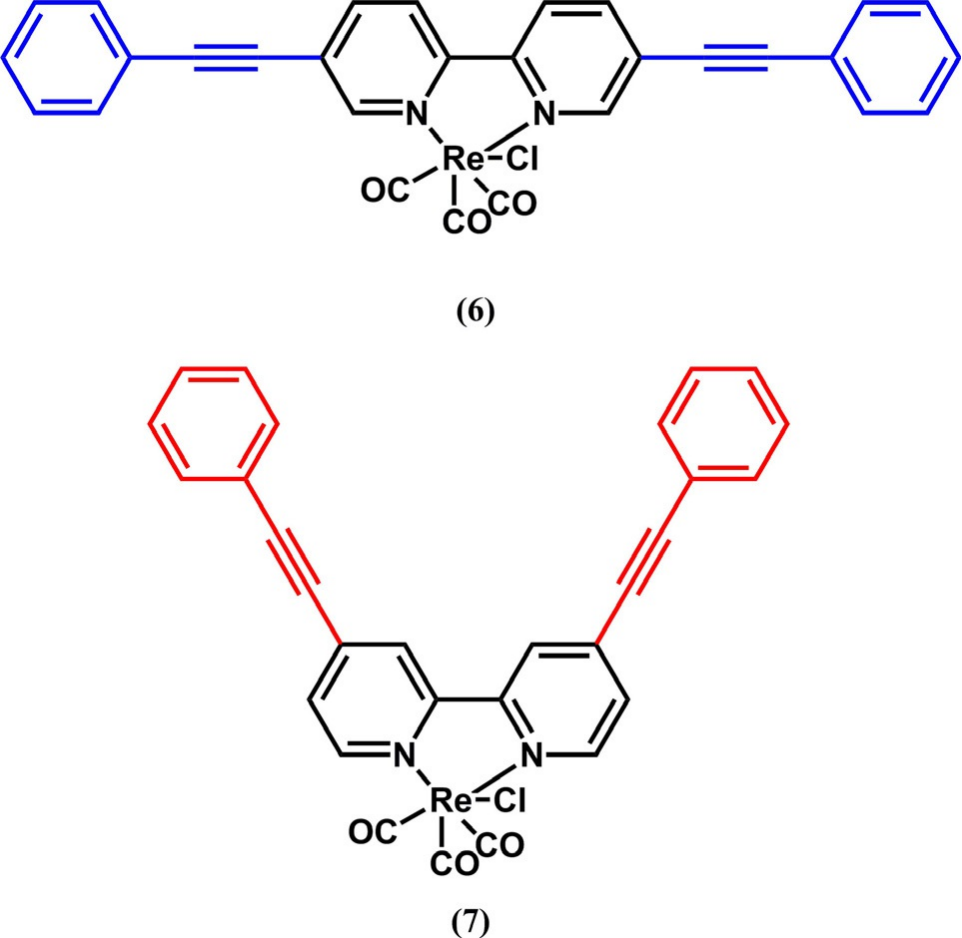
Chemical structures of [{5,5′‐bis(phenylethynyl)‐2,2′‐bipyridyl}Re(CO)_3_Cl] (**6**) and [(2,2′‐bipyridyl)Re(CO)_3_Cl] (**7**).

**Figure 8:**
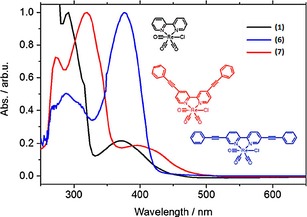
Comparison of normalized UV/Vis absorption spectra of three rhenium carbonyl complexes in acetonitrile solution: **7** (red), **6** (blue), and **1** (black). Reproduced with permission from Ref. 28.

Substitution at the 4‐ and 4′‐positions of the Re‐complexed bipyridyl ligands was shown to be effective by Kubiak and co‐workers.^23^ In that study, Kubiak and his team investigated ligands with different substituents at those positions. Portenkirchner et al. made a comparative study and investigated the differences in catalytic activity arising from the same group substituted at different positions (compounds **6** and **7**, Figure 7).^29^ The authors once again showed the effect of extended π‐conjugation on the absorption characteristics (Figure 8) noting that this can have an impact on photocatalytic properties. Rotating‐disc electrode measurements in this study showed that the diffusion coefficient of **7** was 2.5×10^−6^ cm^2^ s^−1^, which was in good agreement with earlier literature values.^23^, ^30^ If the electrolyte was saturated with CO_2_, compound **7** showed an 11‐fold increase in current at the fourth irreversible reduction at −1600 mV (vs. NHE), which the authors assigned to the metal‐centered reduction. With the information on catalytic current, the authors calculated the catalytic rate constant *k* to be 450 m ^−1^ s^−1^. Using the same method, compounds **1** and **6** yielded *k* values of 60 and 220 m ^−1^ s^−1^, respectively. Despite its higher catalytic rate, compound **7** showed a faradaic efficiency of 12 % after 5 h of electrolysis. The authors explained this phenomenon by the inhibition of catalyst material through side reactions such as dimerization and/or H_2_ evolution. Their report also emphasizes that the nature of the working electrode plays an important role. The authors used two different electrodes—glassy carbon and Pt—noting that if the working electrode was glassy carbon the catalytic rate dropped drastically to 30 m ^−1^ s^−1^. This difference was attributed to the availability of activated protons on the Pt electrode.^29^

Although various molecular catalysts (porphyrins, corroles, cyclams, naphthyridines, and so forth) with metal centers such as Pd, Ru, Fe, Co, and Ni were investigated,^31^, ^32^, ^33^, ^34^, ^35^, ^36^, ^37^, ^38^, ^39^, ^40^, ^41^, ^42^, ^43^, ^44^, ^45^, ^46^, ^47^, ^48^, ^49^ in the years after the discovery of Lehn's catalyst, most research has been focused on Re‐containing complexes. With an estimated average concentration of 1 ppb, Re, together with other noble metals, is one of the rarest elements in Earth's crust. Knowing this fact, Hawecker, Lehn and Ziessel suggested in the outlook of their inspiring work^14^ that the metal center should be substituted with more‐abundant metals such as Mn, Fe, Co, or W. However, up to now there are very few studies in which the Re in bipyridine ligands is substituted.

In 2011, Deronzier and co‐workers introduced a new set of catalysts namely, [Mn(L)(CO)_3_Br] where L is 2,2′‐bipyridine (**1**) or 4,4′‐dimethyl‐2,2′‐bipyridine.^50^ The authors observed two irreversible reduction peaks at around −1600 and −1700 mV (vs. Ag/AgCl), and attributed these peaks to the formation of the dimer [Mn(L)(CO)_3_]_2_ and the mononuclear anion [Mn(L)(CO)_3_]^−^, respectively. They also attributed the oxidation peak around −500 mV to breaking of dimers to give [Mn(L)(CO)_3_(MeCN)]^+^. There was no significant current enhancement if CO_2_ was introduced into solution. However, if there was 5 % H_2_O in the environment, a new peak appeared at a potential that was 150 mV more positive than the first reduction peak and an enhancement of the current was observed. The authors explained this behavior with an analogy to rhenium, in which a weak Brønsted acid such as water was introduced. This helps to stabilize the rhenium–carbon dioxide intermediate and facilitate the cleavage of one of the C−O bonds in CO_2_ to yield CO.^35^ After 22 h of electrolysis authors achieved a faradaic efficiency of 85 % and a turnover number of 34.^50^

In 2013, Kubiak and co‐workers published a detailed follow‐up investigation on the effect of Brønsted acids, including IR spectroscopy results that refine the mechanism further.^51^ They optimized the experimental conditions and obtained faradaic efficiencies approaching unity using Mn complexes. Recently, they improved their results even further by introducing bulky bipyridine ligands to eliminate dimerization reactions. They achieved faradaic efficiencies of 96 % without observing formation of H_2_ as a side product.^52^ These types of catalysts are of great interest not only for carbon monoxide formation but also due to the use of earth‐abundant metals, which might pave the way towards industrial‐scale applications.

### 2.2 Organic Compounds as Homogeneous Catalysts

This part of the Review focuses on the metal free catalysts for electrochemical or photoelectrochemical reduction of CO_2_. To avoid possible confusion, we would like to emphasize that the electrochemical systems described in this part might involve metals as electrodes. However, the focus is on the chemical entity that acts as a catalyst within that specific system.

**Scheme 3:**
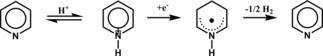
Electrochemical/chemical reactions of pyridine under acidic conditions.

One of the earliest studies that used a metal‐free catalyst was reported by Seshadri, Lin and Bocarsly in 1994.^53^ In this study, the authors utilized a simple molecule, namely pyridine, to perform the electrocatalytic reduction of CO_2_ to methanol. The ionic form of pyridine, the pyridinium cation, acts as the catalyst in this electrochemical system. The authors came to this conclusion by adjusting the pH of the electrolyte to below and above 7. If the pH was greater than 7, no electrochemical features associated with pyridine were observed, indicating that the active species is the pyridinium cation. The optimum pH was found to be 5.4. The authors report that under aqueous conditions pyridinium reduction is coupled via an electrocatalytic electrochemical–chemical mechanism, leading to the reduction of protons to H_2_ (Scheme 3). Although Pd was used as the working electrode, the authors note that this mechanism is valid for other metals using aqueous, nonaqueous electrolytes or mixtures of both.^54^

**Figure 9:**
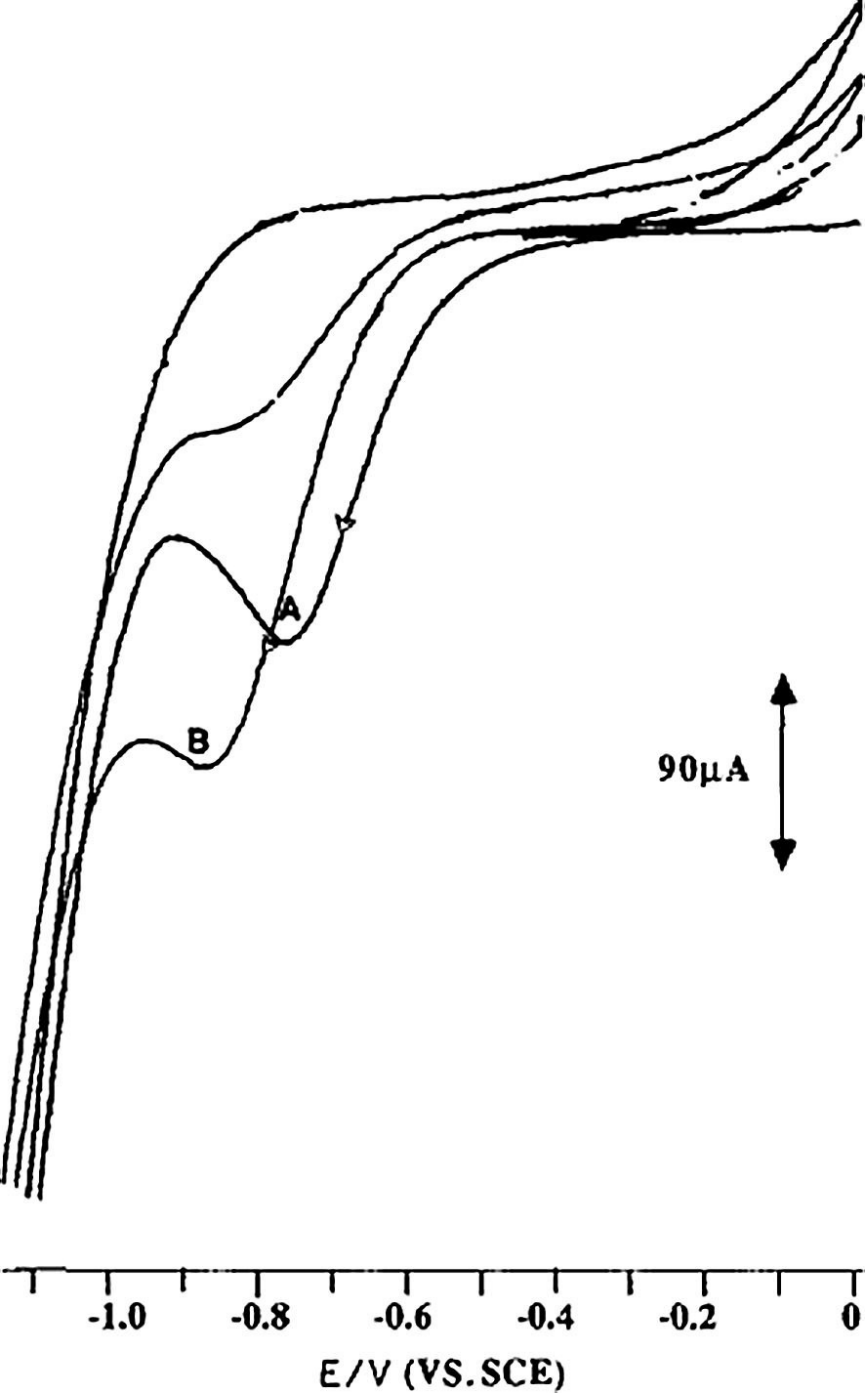
Cyclic voltammograms obtained at a Pd disc electrode (area ≈0.19 cm^2^) in an electrolyte consisting of 0.5 m NaClO_4_ + 10 mm pyridine: under Ar (curve A) and under CO_2_ (curve B). The pH of the solution was maintained at 5.4 for the duration of the experiment with dilute H_2_SO_4_. Reproduced with permission from Ref. 52.

With this reaction mechanism in mind, the authors claimed that the observed current enhancement shown in Figure 9 was indicating an electrocatalytic electrochemical–chemical (EC) mechanism in which dissolved CO_2_ or a species in equilibrium was reduced by the pyridinium ion, delivering pyridine and a reduced CO_2_ species. The authors also performed mass spectroscopy on coulometrically reduced solutions containing CO_2_ and pyridinium, as well as gas chromatography, demonstrating that methanol had formed. Faradaic efficiencies for the methanol production varied between 22–30 % at a constant current of 40 μA cm^−2^ over the course of 19 h on hydrogenated Pd electrodes. The authors also detected formaldehyde as a side product.

**Scheme 4:**
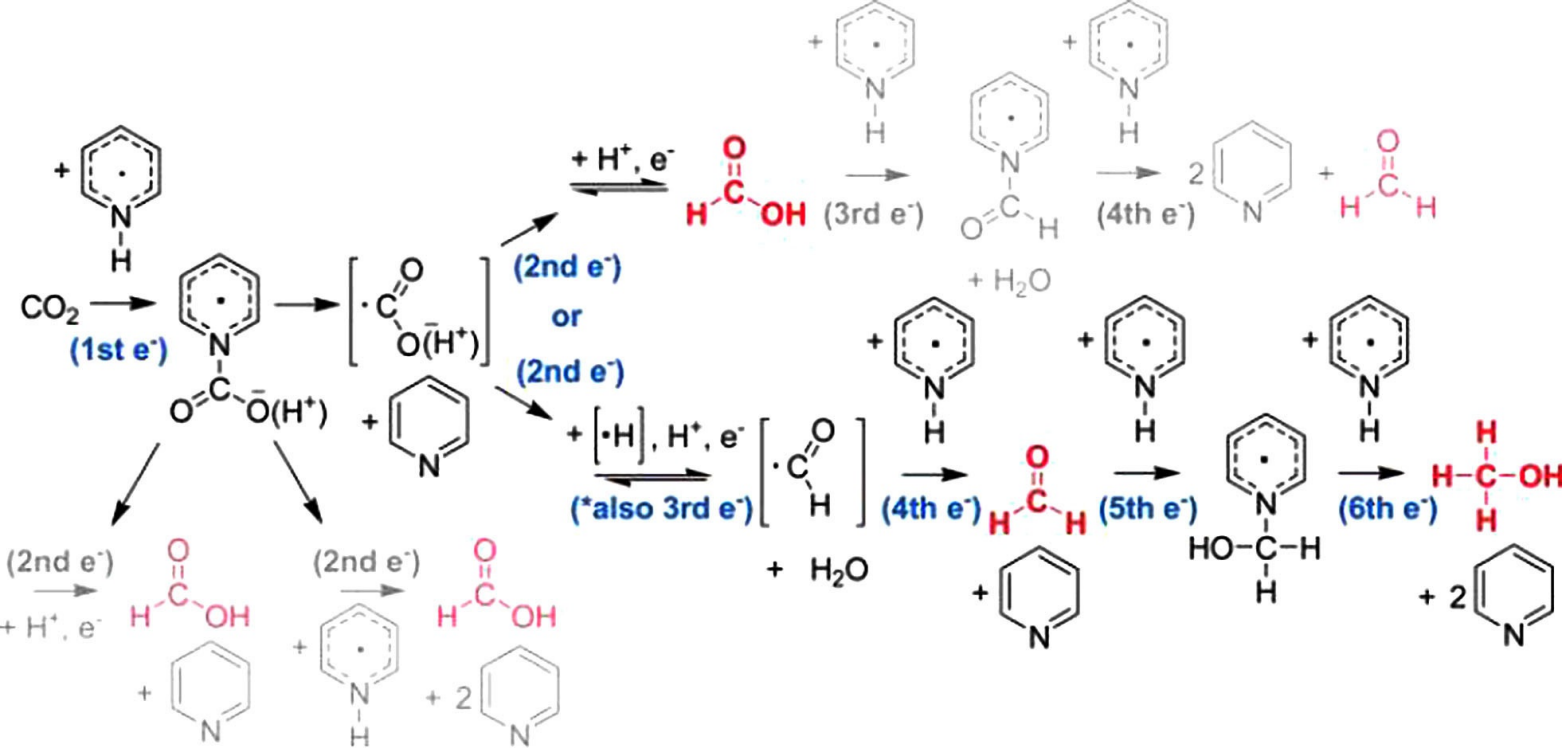
Overall mechanism for the formation of formaldehyde, formic acid and methanol. Reproduced with permission from Ref. 54.

Later in 2010, Bocarsly and his team gave mechanistic insights into how the electrochemical reduction of CO_2_ to methanol proceeds.^55^ They also clarified that formic acid and formaldehyde are the intermediate or side products formed during the electrochemical reduction of CO_2_ to methanol. That reaction requires a proton‐assisted six‐electron reduction with a formal potential of −380 mV (vs. NHE).^55^ According to their findings the mechanism involves multiple single‐electron transfers. Specifically, it was determined to involve two‐electron‐ and four‐electron‐reduced intermediates, which are formic acid and formaldehyde, respectively. The authors elaborated their mechanism by showing all the intermediates and side products as well as the electron flow that leads to methanol formation (Scheme 4).

Authors summarized their findings defining pyridinium radical as the one‐electron charge‐transfer mediator that is responsible for bringing six electrons together to drive electrochemical reduction of CO_2_ to methanol. It was noted that their study is in stark contrast with the common idea of the need of a metal‐based multi‐electron transfer to achieve highly reduced species.

**Figure 10:**
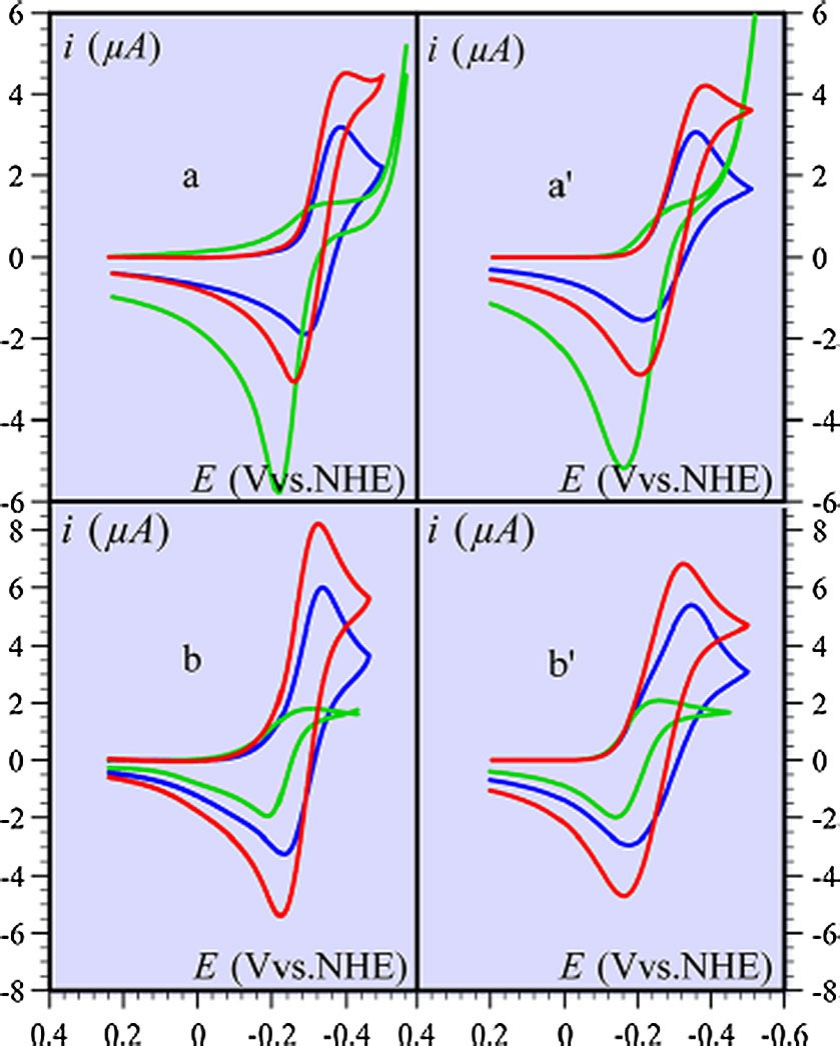
Cyclic voltammograms of a CO_2_‐saturated solution of 3 mm pyridine (panels a and a′) and 3 mm acetic acid (b and b′) on a Pt electrode in the presence of 0.1 m KNO_3_, *T*=295 K, at pH 5.15 (a and a′) and 4 (b and b′). Scan rates: 0.1 V s^−1^ (a and a′) and 0.2 V s^−1^ (b and b′); blue: acid alone; green: CO_2_ alone (pH 4.5 in a and a′, and 4 in b and b′); red: acid + CO_2_. Panels a and b: experimental; a′ and b′: simulations. Reproduced with permission from Ref. 61.

Bocarsly et al. expanded their studies to shed further light on the mechanism. In this study^56^ they concluded that pyridinium is reduced on a Pt electrode, including a pyridinium‐bound proton to form a surface hydride which was further supported by the work of Batista.^57^ Indeed, the study from Bocarsly and co‐workers^55^ created more controversy in the field and drove several studies.^58^, ^59^, ^60^, ^61^ Another study was conducted by Savéant and co‐workers arguing the plausibility of this catalytic process.^62^ The authors argue that the process apparently works only on Pt and/or Pd, metals that are known to reduce hydrated protons.^63^ Their counterargument further continues in two main aspects: challenging the fact that the faradaic efficiency was found to be 20 %,^53^, ^55^ while 100 % of the electrochemical characteristics from cyclic voltammetry were attributed to the process. Second, the authors find confusing the lack of a typical catalytic current enhancement for which the replacement of the peak by a plateau is accompanied by a large increase. To support their argument and for comparison reasons they used acetic acid alongside pyridine. They started off investigating the ion in discussion—pyridinium (PyH^+^)—which is responsible for the catalytic activity. The pH was adjusted to approximately 2 pH units below the p*K*_a_ of PyH^+^ to ensure that the pyridine is in cationic form. The authors observed an increase in the current upon decreasing the pH and they assign this behavior to the reduction of hydrated protons, supported by simulated CVs. The authors suggested that the PyH^.^ radical is not created in the pH range of interest but the electrochemical activity arises from the reduction of hydrated protons generated by the rapid dissociation of PyH^+^ ions. The same phenomenon was observed if pyridine was replaced by acetic acid. Finally, Savéant and co‐workers compared the cyclic voltammograms of pyridine and acetic acid in presence of CO_2_ at pH values adjusted according to their p*K*_a_ values. At a pH at which only the “CE” (chemical reaction followed by an electrochemical one) pathway is dominant, the authors observed an electrochemical behavior arising from the superposition of two acids (PyH^+^ or AcOH and CO_2_) which led them to conclude that PyH^.^ is not formed. They observed the same behavior with acetic acid (Figure 10).

The authors summarized their findings by stating that they did not observe methanol or formic acid formation. In addition, they emphasized that the electrochemical process in question arises solely from the reduction of hydrated protons. Moreover, the formation of a PyH^.^ radical was not observed. At lower pH values the direct reduction of hydrated protons dominates, whereas upon increasing the pH this process is realized through rapid dissociation of acids (PyH^+^ and AcOH).^62^

**Figure 11:**
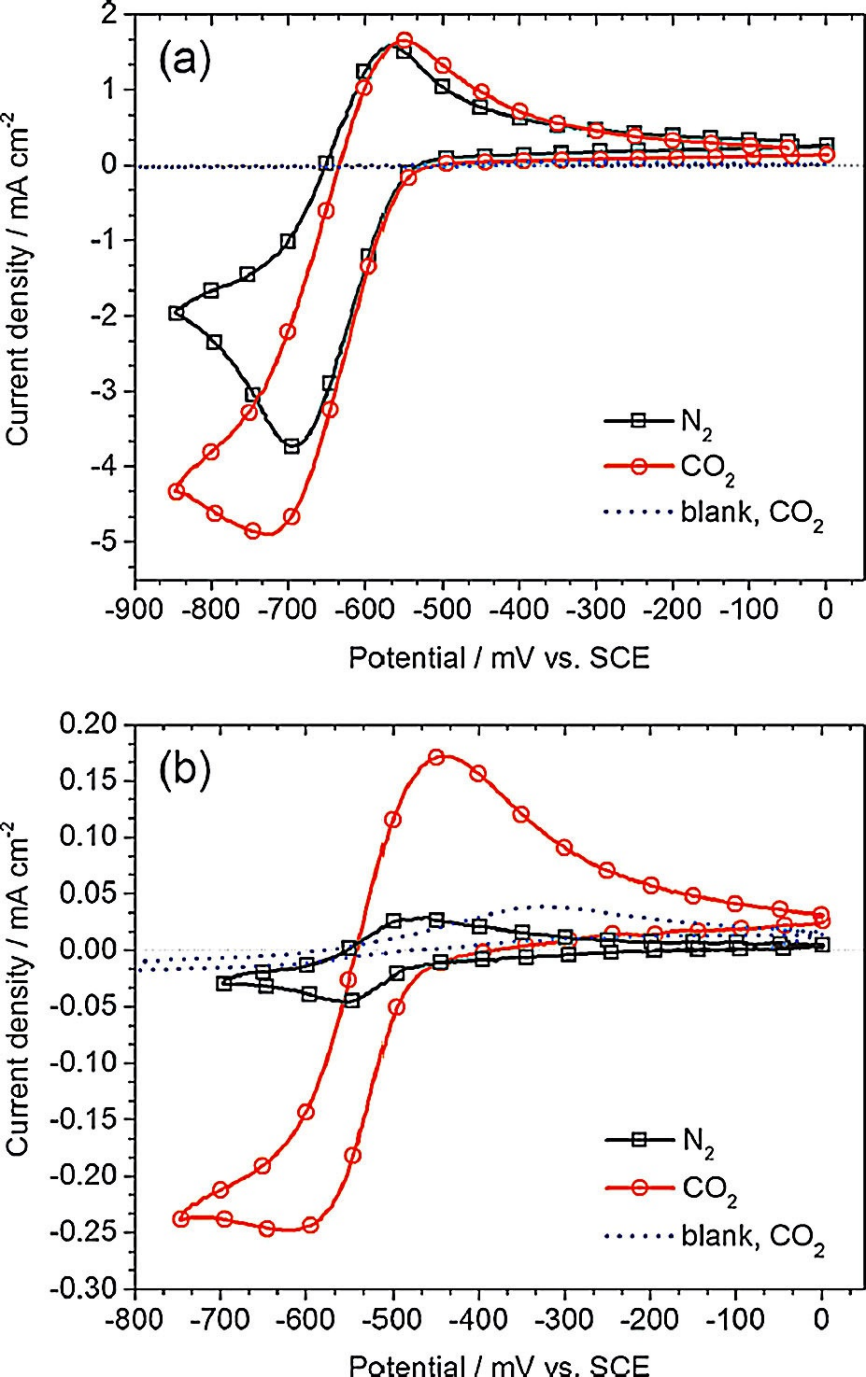
Cyclic voltammograms of a) 50 mm pyridinium in aqueous 0.5 m KCl solution at pH 5.3, and b) 50 mm pyridazinium in aqueous 0.5 m KCl solution at pH 4.7, recorded in N_2_‐ (black line) and CO_2_‐saturated (red line) electrolyte solutions at a Pt working electrode with a scan rate of 25 mV s^−1^. Scans under CO_2_ saturation with no catalyst material present are shown as the blank measurements (blue dashed line). Reproduced with permission from Ref. 63.

Portenkirchner et al. extended the study of pyridinium reduction by introducing pyridazine as a homogeneous catalyst.^64^ An earlier comparison between pyridine and imidazole had also been published.^65^ Despite having a similar chemical structure, the two compounds have quite different p*K*_a_ values: 5.14 for pyridine and 2.10 for pyridazine.^24^ The authors prepared solutions containing 50 mm catalyst material and adjusted the pH to 5.3 for pyridine and 4.7 for pyridazine to obtain their protonated forms. This pH adjustment yielded 0.25 % pyridazinium, which might support the findings of Saveant et al., in which the reduction of parent molecule was not observed and the reductive wave corresponded only to the reduction of hydrated protons created by the rapid dissociation of the parent molecule. The electrochemical behavior of pyridinium and pyridazinium under N_2_‐ and CO_2_‐saturated conditions is shown in Figure 11. A current enhancement of 1.3‐fold was observed for pyridinium if the solution was saturated with CO_2_, while the enhancement was 5.0‐fold in case of pyridazine. An ongoing debate is the effect of the working electrode on the catalytic process. This was addressed in the study by comparing glassy carbon, gold, copper and platinum as the working electrode and only catalytic activity was observed in case of Pt. This was also supported by the work of Musgrave et al. with quantum calculations suggesting that pyridinium was bound to Pt surfaces with an adsorption energy of 1.0 eV per molecule.^66^ The effect of catalyst concentration on the catalytic activity was also studied and the difference in concentration versus current enhancement was compared for pyridinium and pyridazinium. With increasing concentration (from 5 to 100 mm), pyridinium showed a 9.2‐fold increase, whereas the increase with pyridazinium was 1.6‐fold. The authors conducted controlled‐potential electrolysis with solutions containing 50 mm catalyst material for over 30 h. Aliquots of electrolyte solution were analyzed by liquid injection gas chromatography equipped with a flame ionization detector. Methanol and H_2_ were detected as products and the faradaic efficiencies for methanol formation were 14 % and 3.6 % for pyridinium and pyridazinium, respectively. It is also argued in the paper that electrochemical characteristics responsible for the current enhancement might represent a synergetic effect arising from both the catalytic activity and the effects of acids present in the solution, namely pyridinium/pyridazinium and CO_2_. Solutions containing acetic acid did not yield methanol after 30 h of exhaustive electrolysis.

This study adds further evidence supporting the production of methanol using the small molecule pyridine as the electron shuttle to lead a six‐electron reduction of CO_2_ to MeOH.

### 2.3 Enzymes for Homogeneous Bioelectrocatalysis

In considering organic and metal–organic compounds as catalysts, biocatalysts or enzymes also have to be taken into account. Enzymes are proteins with an active site, responsible for catalyzing diverse reactions. In the case of CO_2_ reduction, dehydrogenase enzymes have especially gained interest as they are capable of reducing CO_2_ to formic acid, formaldehyde, methanol, or carbon monoxide. The use of enzymes to drive electrocatalytic reduction of CO_2_ has advantages. Firstly, due to electrostatic interactions enzymes tend to retain the most favorable structure, exposing a maximum amount of catalytically active sites, which in turn increases the probability of enzyme–substrate interactions (CO_2_ is the substrate in this case). Secondly, by definition, enzymes have high selectivity towards certain products, and side reactions are suppressed.

The report from Höpner and co‐workers^67^ is considered to be one of the earliest studies on enzymatic CO_2_ reduction after the discovery by Stephenson and Stickland showing the synthesis of formic acid from H_2_ and CO_2_ using *E. coli* cells.^68^ The authors used nicotinamide adenine nucleotide (NADH) as the reducing agent for catalyzing the reduction of CO_2_ with formate dehydrogenase (FDH) to yield HCO_2_^−^, which is considered to be one of the safest methods to store hydrogen.^69^, ^70^ They also showed that the source of carbon in formate was indeed CO_2_ using experiments with ^14^C. In 1982, Klibanov and co‐workers published a study showing enzymatic production of formic acid using H_2_ and CO_2_ with different electron carriers.^69^ The authors also introduced a method for utilizing formic acid to produce H_2_ gas.

Although these early papers did not address the electrochemical reduction of CO_2_, they were the pioneering studies that planted the idea of using enzymes as catalysts. Although enzyme‐catalyzed CO_2_ reductions provide sustainable pathways with high selectivities and yields of generated products, the application of biocatalysts is limited to laboratory‐scale experiments. Due to the necessity of sacrificial co‐factors such as NADH, those processes are limited, as synthesis and regeneration of co‐factors is expensive. Substitution of co‐factors as electron donors by photochemical, photoelectrochemical or electrochemical strategies therefore became highly attractive.^71^

**Scheme 5:**
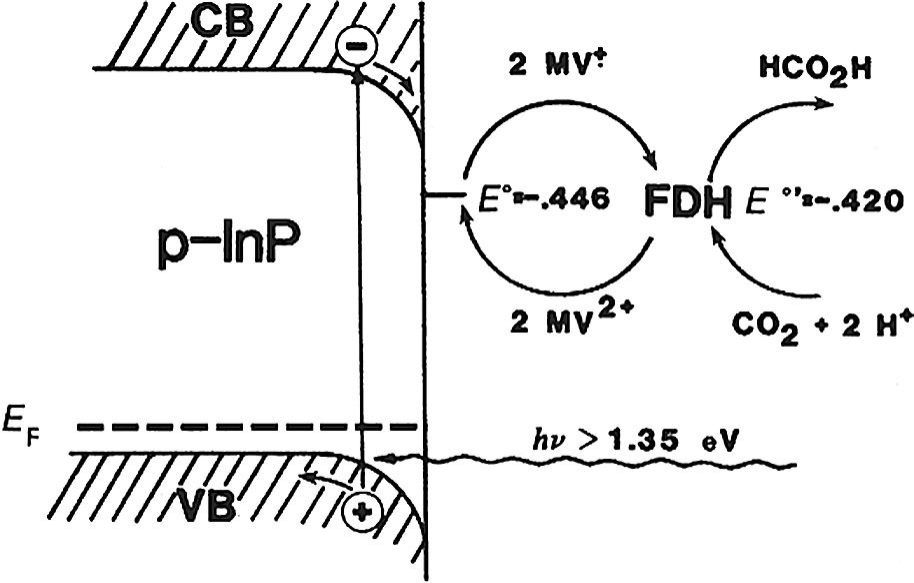
The photoelectrochemical production of CO_2_ with the enzyme formate dehydrogenase (FDH) as the catalyst and methyl viologen (MV^2+^) as the mediator. p‐InP: p‐type indium phosphide; VB: valance band; CB: conduction band. Reproduced with permission from Ref. 71.

Parkinson and Weaver reported the electrochemistry of an enzyme using a mediator for the first time in 1984.^72^ The authors described the use of a semiconductor, p‐type indium phosphide (p‐InP), together with FDH to yield formic acid using CO_2_. This was a photoelectrochemical system using the p‐InP electrode as the source of photogenerated electrons and methyl viologen (MV^2+^) served as the electron shuttle for the FDH. The mechanism for the CO_2_ reduction is shown in Scheme 5. The potentials given in the scheme represent the formal redox potentials of MV and FDH, respectively. Upon irradiation, an electron is excited to the conduction band, whereas the hole in the valence band is extracted by the electrode and the electron in the conduction band is transferred to MV^2+^, converting it to MV^+^. The authors obtained a faradaic efficiency of 89 % and a TON of 21 000 using their system.^69^ This study was important in the sense of removing the need for an expensive electron donor, NADH, for the conversion of CO_2_ to formate and replacing it with photogenerated electrons.

Kuwabata, Tsuda and Yoneyama later used FDH and methanol dehydrogenase (MDH) to convert CO_2_ to formic acid and methanol, respectively.^73^ They used MV^2+^ or pyrroloquinoline quinone (PQQ) as an electron mediator in buffered solutions applying potentials ranging from −700 to −900 mV (vs. SCE) and achieved faradaic efficiencies of 90 %. Small amounts of formaldehyde were also observed. The authors found out that methanol production begins only if the formaldehyde concentration reaches 1 μm in the electrolyte solution. The authors concluded that upon changing the concentration of both MDH and the mediator MV^2+^, the formation of methanol proceeds through formate and formaldehyde consecutively. The faradaic efficiency for methanol was calculated as 45 %. If the electron mediator was changed from MV^2+^ to PQQ there was a remarkable change in the faradaic efficiency for methanol production. The authors did not observe the presence of formaldehyde in the electrochemical cell, whereas the amount of methanol reached 1.4 μmol after a certain initial period, yielding a faradaic efficiency of 89 %.^73^ This suggests a different mechanism for the formation of methanol that does not involve formaldehyde as an intermediate. However, the authors did not comment on the mechanism in their study.

**Figure 12:**
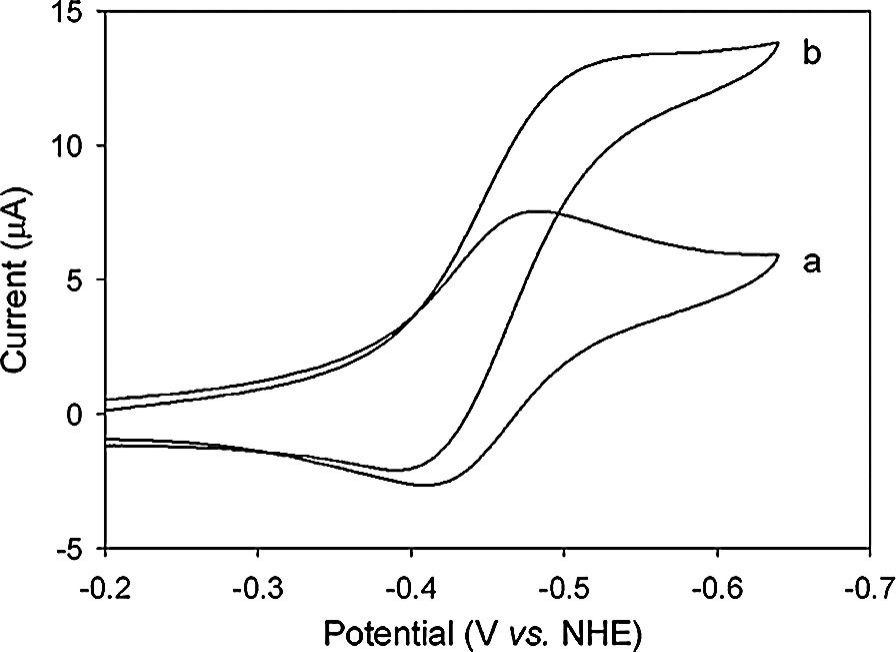
Cyclic voltammograms of a) 1.0 mm MV^2+^ in 0.1 m phosphate buffer (pH 7.0), and b) 1.0 mm MV^2+^ containing 1 atm CO_2_‐saturated 0.1 m phosphate buffer (pH 6.3) with 0.5 mg mL^−1^ of carbon monoxide dehydrogenase at 50 °C. A glassy carbon disc (3 mm diameter) was used for the working electrode, scan rate 10 mV sec^−1^. Reproduced with permission from Ref. 73.

Kim and co‐workers used carbon monoxide dehydrogenase (CODH) to drive an electroenzymatic reduction of CO_2_.^74^ In their report, the authors note that MV^2+^ was also required to address the enzyme electrochemically, whereby they used a glassy carbon disk as the working electrode. They achieved a faradaic efficiency reaching unity at thermodynamic potentials (−570 mV vs. NHE) with a TON of 700. The high faradaic efficiency was attributed to the highly selective binding of CO_2_ to the enzyme active center which contains Ni and Fe atoms. The electrochemical behavior of MV^2+^ and CODH in solution was investigated using cyclic voltammetry (Figure 12).

The authors argued that the electrochemical wave observed upon saturating the solution with CO_2_ (Figure 12, curve b) is an indication of the electrochemical reduction of CO_2_ to CO by CODH. They explain the lack of catalytic enhancement in the current with the high formal reduction potential of direct CO_2_ reduction. However, they do not show any spectroscopic and/or electrochemical data to confirm the function of the enzyme in the solution using MV^2+^.

Another study was conducted using *Candida boidinii* formate dehydrogenase (cbsFDH), which is an enzyme requiring electrons, protons and NADH to drive CO_2_ reduction to formic acid. MV^2+^ was used as electron shuttle yielding 24 % faradaic efficiency.^75^ NADH is an expensive material to use as the electron source and needs to be regenerated. For that reason, authors introduced an Rh complex, [Cp*Rh(bpy)Cl]^+^. Using cbsFDH, the authors increased selectivity towards formate without having HCO_3_^−^ as a side product.

**Figure 13:**
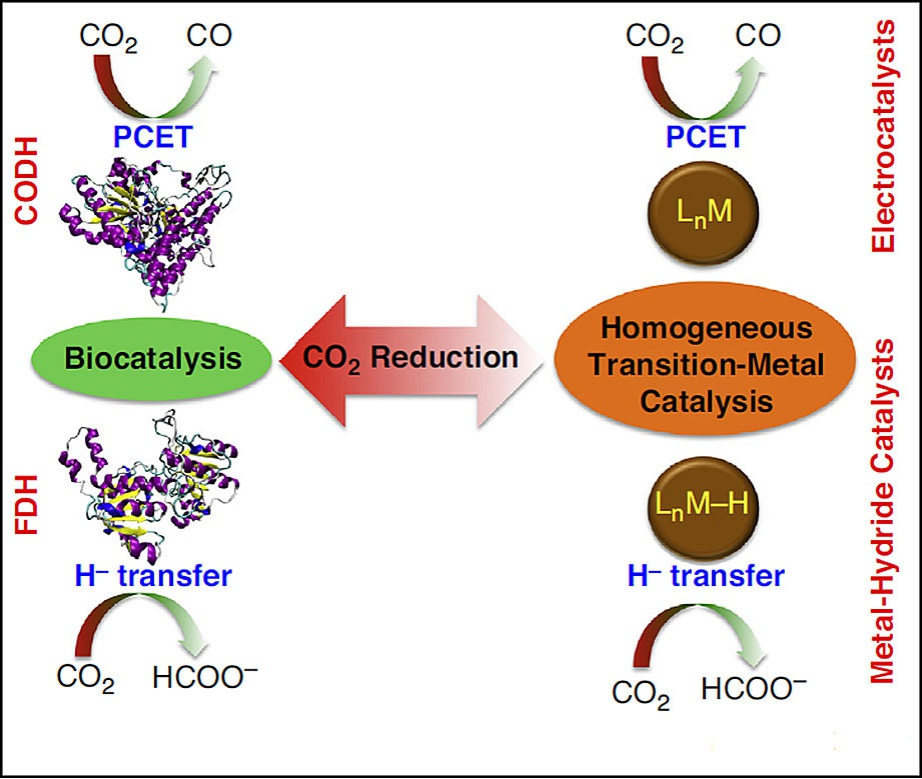
Overview of the bio‐inspired mechanism for the two‐electron reduction of CO_2_ to CO and formate (PCET=proton‐coupled electron transfer). Reproduced with permission from Ref. 70.

Similar studies can be found in the literature in which the effects of different enzyme types having different metal active centers and the contribution of those metals to the formation of CO and formate were investigated.^76^, ^77^ Nørskov and co‐workers reported a good overiew of the design parameters for enzymatic catalysts using DFT calculations. One of the important points noted in their paper is the ability of the metal center to bind CO and HCOO^−^ while stabilizing oxygen in the latter. They also suggest that readers use noble metal centers in enzymes as such metals have rather high overpotentials for H_2_ formation.^76^ In their review, Mondal et al. give mechanistic insights into CO_2_ reduction and in particular, the mechanistic aspects of its reduction to CO compared to formate (Figure 13).^71^

## 3 Heterogeneous Electrocatalysis for CO_2_ Reduction

Homogeneous approaches for CO_2_ reduction have been widely used throughout the history of the field. However, as described in the previous section, electrochemical addressing of the catalyst material, which is distant from the electrochemical double layer might be an issue. Also, recovery of the catalyst or separation of the product from the homogeneous mixture is a technical problem. Different mechanisms can lead to degradation, inhibition, and eventual decrease in overall efficiency. Bearing these drawbacks in mind, researchers have focused on the direct use of catalysts using the idea of heterogeneous catalysis. The following sections focus on heterogeneous ap‐ proaches towards electrochemical and photoelectrochemical CO_2_ reduction.

### 3.1 Catalyst‐Functionalized Metal/Metal‐like Electrodes

A study by Lieber and Lewis was one of the earliest that investigated a heterogeneous approach.^78^ Pyrolytic graphite or carbon cloth were used as electrodes and they were modified with cobalt phthalocyanine, Co(Pc), via drop‐casting or adsorption from solutions of Co(Pc) in THF. The authors conducted cyclic voltammetry measurements in aqueous citrate buffer (pH 5) with and without a CO_2_ atmosphere. Interestingly, there was no change in the voltammetric behavior of Co(Pc) surfaces in a CO_2_ atmosphere. The authors argued that the initial step did not involve binding of CO_2_ on the reduced species Co(Pc)^−^. However, they did not exclude the possibility of very weak binding. From the results of experiments at different pH values, the authors concluded that the mechanism involves an initial protonation of Co(Pc), followed by binding of CO_2_. The authors reported the observed products as CO (major product), H_2_ and formate/oxalate (trace amounts). They achieved faradaic efficiencies of up to 60 % for CO and 35 % for H_2_. A TON of 370 000 was achieved, which represented an improvement over the previously reported result by three orders of magnitude.^31^

**Figure 14:**
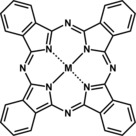
Chemical structure of metal phthalocyanine.

In 1989, Furuya and Matsui reported their findings on the efficiency of 16 different metal phthalocyanines (Figure 14) in the electrochemical reduction of CO_2_.^79^ They focused on different transition metal groups, namely groups VIII, IIIB and IVB. Phthalocyanines were immobilized on gas‐diffusion electrodes, which were prepared using hydrophobic carbon black, hydrophilic carbon black, and polytetrafluoroethylene. The electrolyte solution was saturated with CO_2_ and the cathode (gas diffusion membrane–phthalocyanine) was purged constantly with CO_2_ and H_2_ from the back side of the electrode. Electrolyses were conducted under galvanostatic conditions with 0.5 m KHCO_3_ serving as the electrolyte. Table 1 summarizes the metals used, the main products formed and corresponding faradaic efficiencies achieved.

**Table 1:** List of phthalocyanines used (according to their metal centers), products and their faradaic efficiencies.

Metal center	Product	Faradaic efficiency [%]
Co, Ni	CO	≈100
Sn	HCOOH	70
In	H2	65
Pb	HCOOH	65
Cu, Ga, Ti	CH4	30–40
Fe, Pd	CO	50–80
Zn	CO	15
Al	HCOOH	15
Pt, Mg, V, Mn	H2	≈100

The authors also provide insight into the probable mechanisms leading towards the products. For CO formation, the authors suggest coordination of CO_2_ to the metal center to be the first step. Addition of two hydrogen atoms to the metal‐coordinated CO_2_ was proposed to be the initial steps of formic acid formation. Finally, methane was proposed to form by elimination of an oxygen atom after formation of CO. Mechanistic details and a step‐by‐step breakdown of the reactions are reported in the paper.^79^

**Scheme 6:**
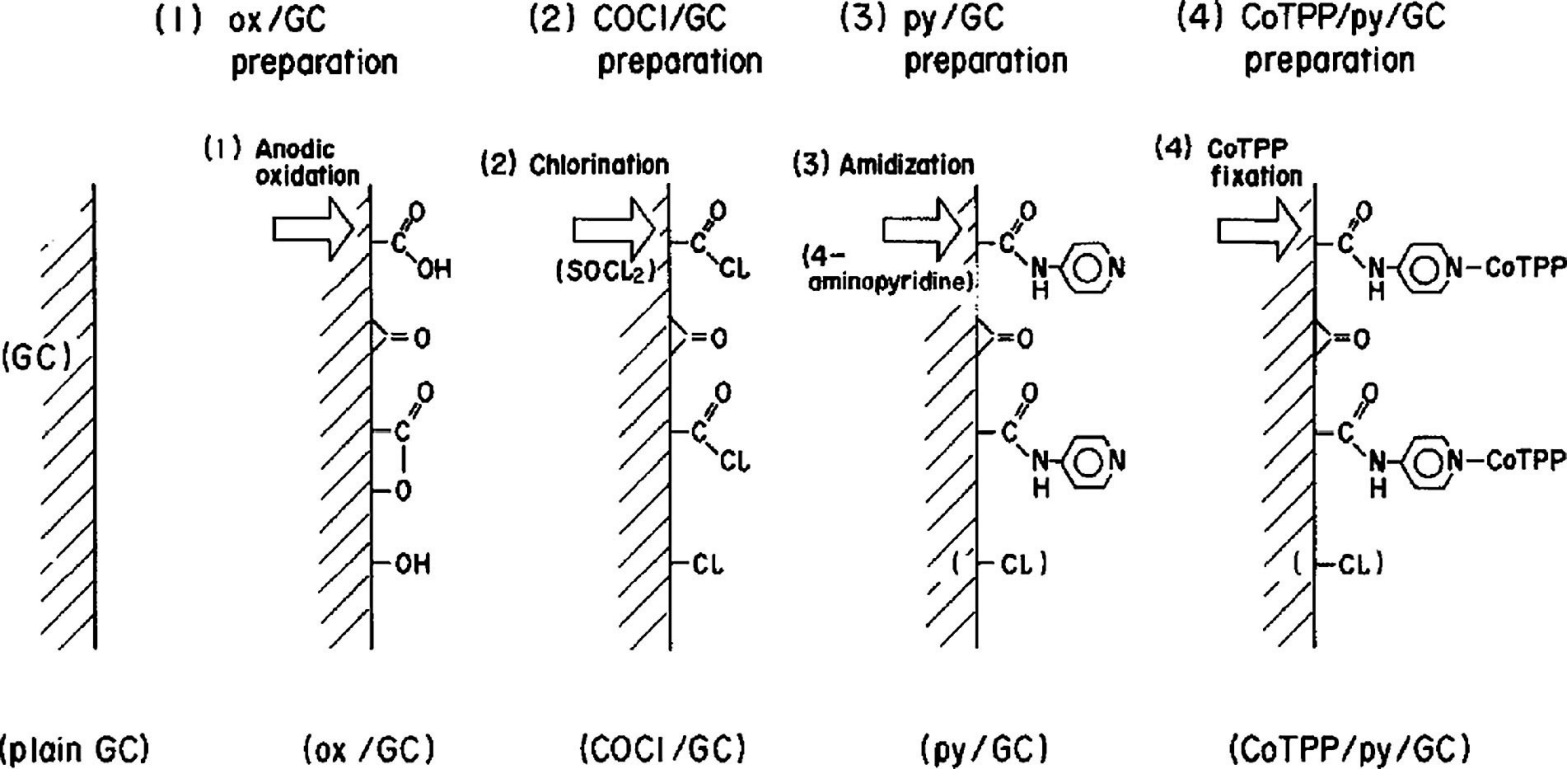
Procedure for the preparation of CoTPP–pyridyl–glassy carbon electrode. Reproduced with permission from Ref. 79.

Another approach in which macrocycles were used as catalysts came from researchers at Hokkaido University in 1991. Enyo and co‐workers immobilized cobalt(II) tetraphenylporphyrin (CoTPP) using pyridine as the chemical anchor on a glassy carbon surface.^80^ Preparation of modified glassy carbon electrodes is shown in Scheme 6. CO was detected as the only product after constant‐potential electrolysis at potentials from −1000 to −1300 mV (vs. SCE). The authors observed the optimal potential at −1100 mV and achieved a faradaic efficiency of 92 and a remarkable TON of 10^7^. To assess the effect of fixation on the stability, the authors conducted constant‐potential electrolysis experiments under homogenous conditions in which they dissolved CoTPP and pyridine in a solution of tetra‐*n*‐butylammonium fluoride in DMF. Catalytic activity degraded rapidly when the potential was applied and a black precipitate was observed at the bottom of the electrolysis cell, whereas the modified electrodes showed constant catalytic activity for up to 5 days. The authors explained the stability of the electrodes and hence the stability of catalytically active CoTPP with the presence of the pyridine ligand, which introduces isolation of CoTPP centers from each other. The second improvement from the pyridine ligand is the introduction of vacant sites for CO_2_ due to the direction effect by the ligand.^80^

Yoshida and co‐workers contributed study of immobilized catalysts by introducing catalysts that were incorporated into coated Nafion membranes. Coated Nafion served to create a hydrophobic environment around the catalyst to suppress hydrogen evolution.^81^ The catalysts used were [Re(bpy)(CO)_3_Br] and [Re(terpy)(CO)_3_Br] (terpy=2,2′:6′,2“‐terpyridine). Basal‐plane pyrolytic graphite (BPG) was used as the electrode, which was coated with Nafion containing 2.6 mmol catalyst material. Constant‐potential electrolysis resulted in a variety of products (HCOOH, CO and H_2_), formic acid being the main one. A maximum faradaic efficiency of 48 % was achieved for formic acid with a TON of 98 with [Re(bpy)(CO)_3_Br] as the catalyst in the Nafion matrix. Higher TONs were achieved for CO production, the highest was 198.^81^

The same group also reported the immobilization of a CoPc–poly(4‐vinylpyridine) matrix on a pyrolytic graphite to achieve CO_2_ reduction with CO as the main product. The faradaic efficiencies varied between 31 % and 43 %, with remarkable TONs of around 10^5^.^82^ Interestingly, in the years that followed, there have not been many studies on catalyst immobilization on metal or metal‐like surfaces.

**Figure 15:**
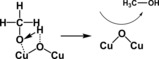
Hydrogenation of methoxy adsorbates at Cu_2_O surfaces.

Copper has always been the choice of metal if several products and higher hydrocarbons such as methanol, methane, propanol, or formic acid were desired. Readers are advised to read the detailed work of Hori et al. on the electrochemical reduction of CO_2_ using various metals.^83^ Flake et al. reported their findings on copper oxide as a catalyst for electrochemical CO_2_ reduction.^84^ They used copper foils as the electrodes, which formed cuprous oxide (Cu_2_O) on the surface. The electrodes were tested in 0.5 m KHCO_3_ electrolyte for their performance as CO_2_ reduction catalysts. Methanol was the major product after 30 min electrolysis at −1100 mV (vs. SCE) with a faradaic efficiency of 38 %. If the potentials exceeded −1550 mV (vs. SCE), there was a rapid decrease in methanol production and H_2_ evolution prevailed. Also, electrolysis durations of more than 30 min yielded methane as the main product. This behavior can be explained by the reduction of Cu^I^ species over time to Cu^0^ to give methane. Another important point demonstrated by the authors was that the mechanism goes through H_3_CO^−^ species (Figure 15).^84^ Other studies using Cu_2_O films as well as catalysts having core–shell structures have been reported for similar materials with comparable efficiencies.^85^, ^86^

Recently, researchers from the University of Liverpool adapted the study of Yoshida and co‐workers to use [Mn(bpy)(CO)_3_Br]. They immobilized the catalyst in a matrix covering a glassy carbon electrode.^87^ The electrochemical characteristics of the compound were in agreement with previous studies.^50^, ^51^ Using this compound, the authors obtained CO and H_2_ as products. Formation of the primary product was dependent on the potential applied. For example, if −1500 mV (vs. Ag/AgCl) was applied, CO was the main product with a faradaic efficiency of 51 %. If the potential was switched to −1600 mV (vs. Ag/AgCl) H_2_ evolution dominated with 81 % faradaic efficiency. It was noted that use of glassy carbon, which has a smooth texture, leads to lower concentrations of the catalyst material in the Nafion matrix. To fix this issue, the authors included multiwalled carbon nanotubes (MWCNTs) for increased surface area and therefore an increased catalyst concentration. A 10‐fold current increase was observed under CO_2_‐saturated conditions if MWCNTs were introduced; however, H_2_ was the main product instead of CO.^87^ For some time, Nafion was the material of choice for fixing electrocatalysts onto electrodes. The idea is to have a hydrophobic environment around the catalyst and thus minimize H_2_ evolution to a certain extent. However, this prevented the access of the electrolyte solution into the matrix in the case of an aqueous environment.

**Figure 16:**
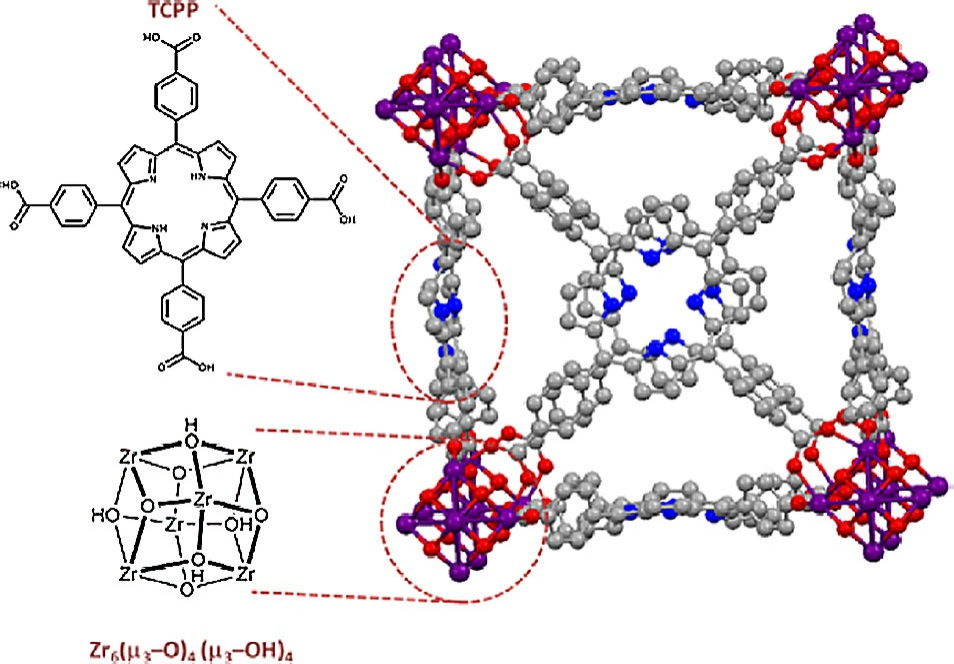
Illustration of a portion of the crystal structure of MOF‐525 in porphyrin free‐base form, including the chemical structure of the TCPP linker and the Zr_6_‐based node. Reproduced with permission from Ref. 87.

Hupp and co‐workers formulated the idea of incorporating a known catalyst, that is, iron tetracarboxyphenylporphyrin (Fe–TCPP), into a metal–organic framework (MOF).^88^ The authors note that the choice of MOF (Figure 16) facilitated the access of solvent, reactant and electrolyte solution further into electroactive sites. Furthermore, the metalloporphyrin linkers within the MOF served as both electrocatalysts and as redox‐hopping moieties for the delivery of reducing equivalents to the catalytic sites. The authors achieved faradaic efficiencies of up to approximately 60 % for CO production.

**Figure 17:**
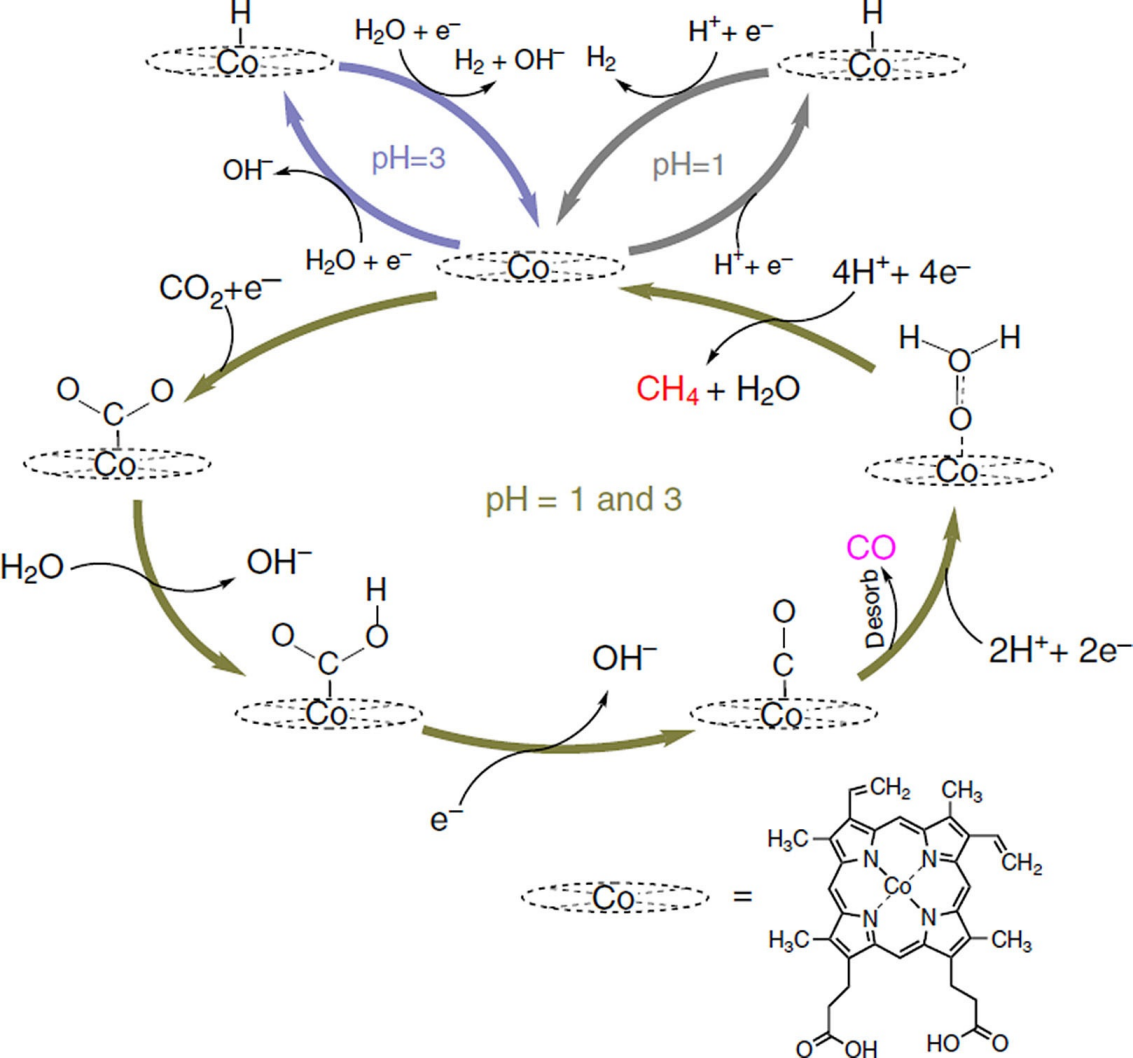
H^+^ and H_2_O are the hydrogen sources for the hydrogen evolution reaction at pH 1 and 3, respectively. CO_2_^.−^ is the initial intermediate for the reduction of CO_2_ to CO. CO can be further reduced to methane with HCHO as an intermediate. The catalytically inactive “resting” state of the Co is assumed to be 2+. The reduction of Co^2+^ to Co^+^ is supposed to trigger both the H_2_ evolution and CO_2_ reduction pathways. Reproduced with permission from Ref. 88.

The majority of studies in which porphyrins and phthalocyanines were used as catalysts did not include detailed mechanistic accounts. Koper and co‐workers utilized in situ measurement techniques such as online electrochemical mass spectroscopy and online HPLC to address this issue.^89^ In their study, the authors used cobalt protoporphyrin as the electrochemical catalyst, which they immobilized on a pyrolytic graphite electrode. CO and CH_4_ were the two main products. Methane production was achieved by reducing CO with HCHO as an intermediate. The mechanism proposed by the authors is given in Figure 17.^89^

### 3.2 Catalyst‐Functionalized Semiconductor Electrodes

This part of the Review is focused on the direct use of semiconductor electrodes for reduction of CO_2_ as well as catalyst‐functionalized semiconductor electrodes.

One of the early studies from Halmann used p‐type gallium phosphide (GaP) as the photoelectrode for driving the reduction of CO_2_.^90^ GaP was immersed in an electrolyte solution together with a graphite rod as the counter electrode and a saturated calomel electrode as a reference electrode. The choice of counter electrode was a strategic decision, as it was reported that carbon oxidizes neither formic acid nor carbohydrates to carbon dioxide.^91^ The GaP electrode was illuminated using a Hg arc lamp and was biased with −1000 mV (vs. SCE). Analysis of the electrolyte revealed the presence of formic acid, formaldehyde and methanol at concentrations of 1.2×10^−2^, 3.2×10^−2^ and 8.1×10^−4^ m, respectively, after 18 h of irradiation.^90^ In 1983, another study in which GaP was used for photoelectrochemical reduction of CO_2_ reported faradaic yields of 15.2 % for HCOOH and the influence of the pressure on the reduction of CO_2_ was investigated.^92^

The use of semiconducting electrodes for reduction of CO_2_ was favored in the years that followed. Canfield and Frese published their findings on the use of GaAs and InP as the catalytic materials. They used n‐type GaAs to drive the electrocatalytic reduction of CO_2_ to methanol with a faradaic efficiency of 89 %, whereas p‐type GaAs and InP were used as photoelectrocatalysts to yield methanol with faradaic efficiencies of 52 % and 80 %, respectively.^93^

Building on their own studies,^53^ Bocarsly and co‐workers reported the use of a p‐type GaP semiconductor as the electrode and pyridinium as the catalyst. The conditions they used were almost identical for pyridinium except the electrode was GaP instead of hydrogenated Pd. They achieved faradaic efficiencies up to 96 %.^94^ The use of semiconductors as electrodes and catalysts in solution was investigated by several groups. These studies yielded a range of products, from CO and H_2_ 95, 96, 97 to methanol.^98^

**Figure 18:**
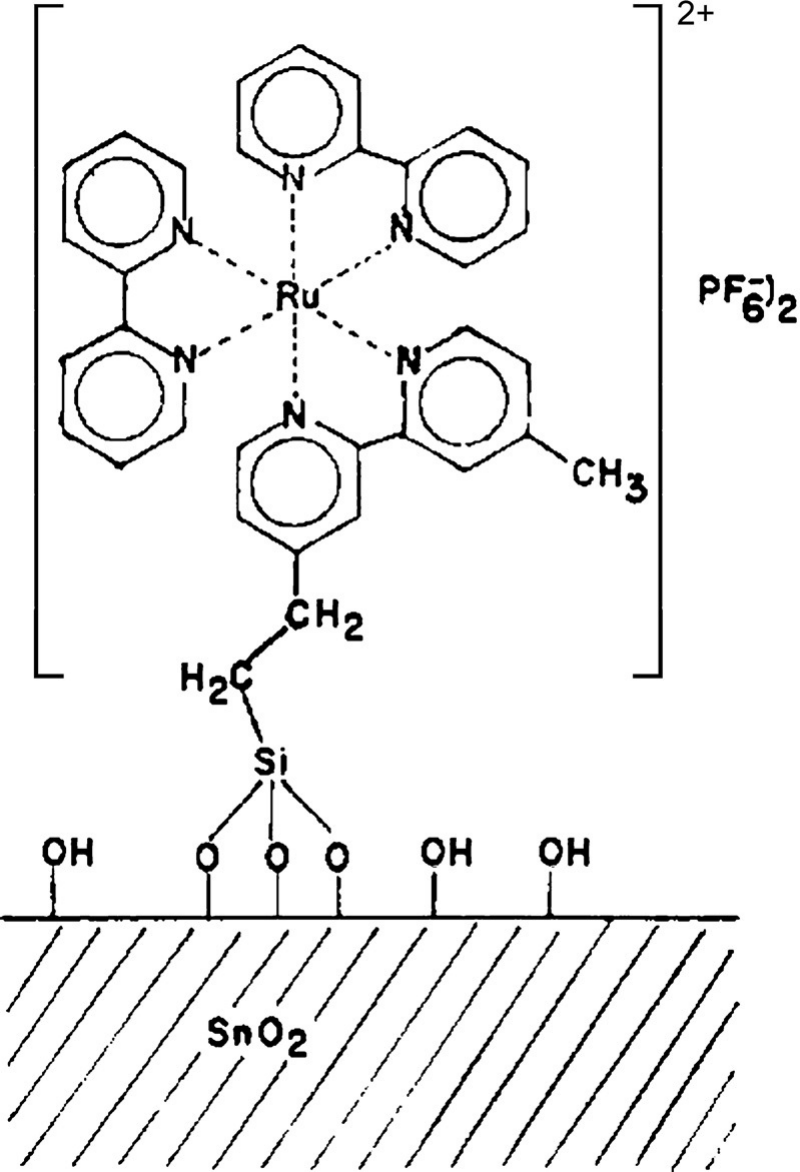
Covalently attached [Ru(bpy)_3_]^2+^ on a SnO_2_ surface. Reproduced with permission from Ref. 98.

Several other studies focused on immobilization of catalytically active materials on semiconductor surfaces. Ghosh and Spiro reported their results on covalent immobilization of [Ru(bpy)_3_]^2+^ on SnO_2_ surface (Figure 18).^99^ Although they did not investigate the catalytic activity of the Ru complex, this was an important study in terms of immobilization of such compounds on semiconductor surfaces and in terms of their electrochemistry and photoelectrochemistry.

**Scheme 7:**
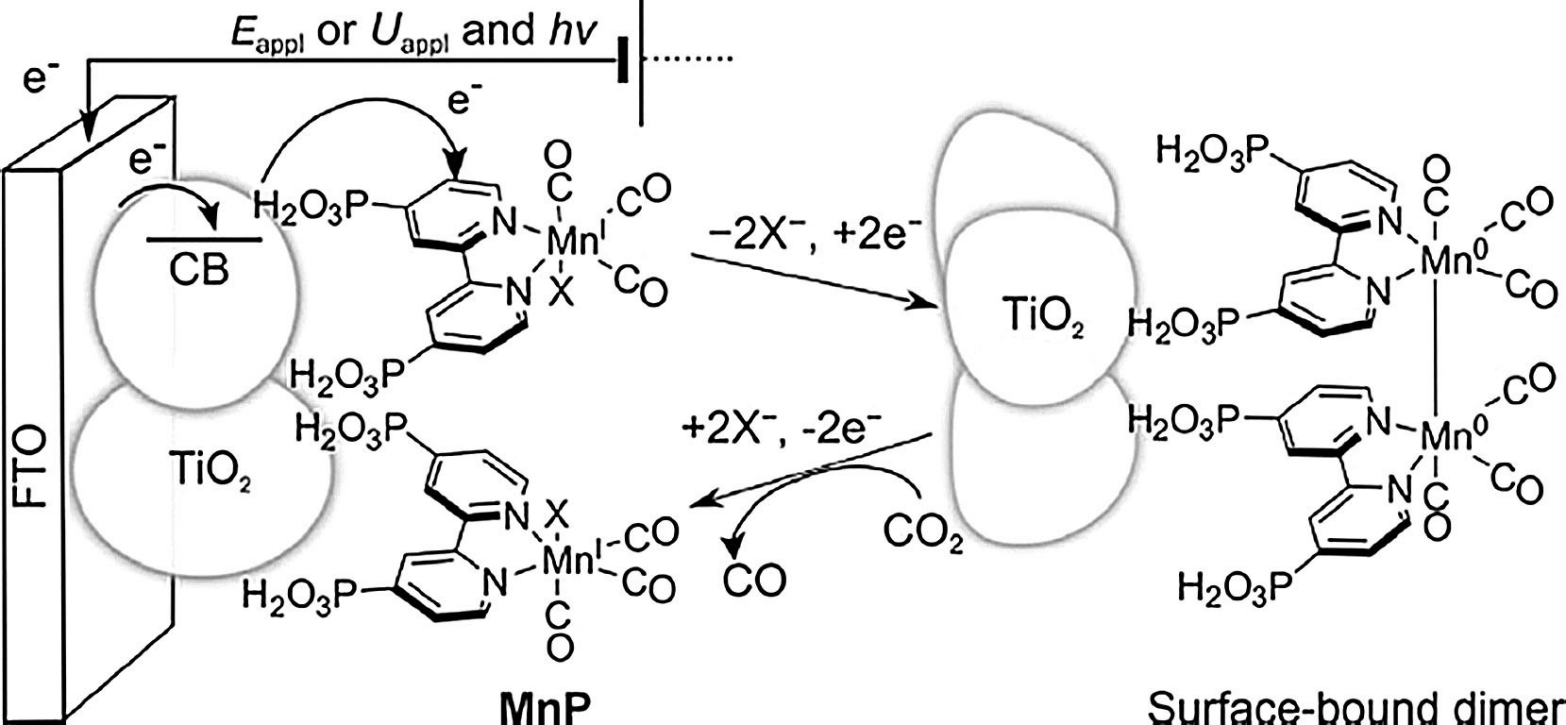
Illustrated proposed mechanism for CO_2_ reduction by TiO_2_–MnP (X=Br^−^ in the isolated compound). Reproduced with permission from Ref. 100.

Reisner and co‐workers immobilized modified Re complexes, namely [Re{2,2′‐bipyridine‐4,4′‐bis(phosphonic acid)}(CO)_3_(L)] (L=3‐picoline or bromide) on TiO_2_ nanoparticles.^100^ They used these modified structures as photocatalysts for reducing CO_2_ to CO, achieving a TON of 48 mol_CO_ mol_Re_^−1^. In the following year, the same group reported their findings on immobilized Mn complexes using the same ligand mentioned above to yield [Mn{2,2′‐bipyridine‐4,4′‐bis(phosphonic acid)}(CO)_3_Br] (MnP).^101^ MnP was immobilized on mesoporous TiO_2_ by drop‐casting and the amount of catalyst was calculated as 34 nmol cm^−2^. The authors claim that the phosphonic acid groups act as the anchoring moieties. They achieved a TON of 112 at moderately low overpotentials of 420 mV, with a faradaic efficiency of 67 %. The authors proposed mechanism for the process via immobilized MnP is shown in Scheme 7, and they noted that use of such immobilization techniques will help to improve long‐term stability and conductivity under reducing conditions. In addition, the presence of a 3D structure would increase the catalyst loading as well as facilitate intermolecular interactions.^101^

### 3.3 Catalyst‐Functionalized Organic Semiconductor Electrodes

This section is focused on catalyst‐functionalized electrodes for the heterogeneous electrochemical/photoelectrochemical reduction of CO_2_. The term “organic semiconductor electrodes” denotes that the electrons are transferred via the organic semiconductor to the catalyst material or to CO_2_, although it does not mean that the electrode itself is a free‐standing organic semiconductor structure.

**Figure 19:**
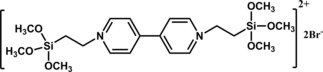
Chemical structure of a bipyridine monomer.^102^

Wrighton and co‐workers reported the immobilization of Pd in a bipyridine‐based polymer, (PQ)^2+^, and its catalytic activity for reduction of HCO_3_^−^ to HCO_2_^−^ in the presence of H_2._ 102 The authors first polymerized the bipyridine monomer (Figure 19) on tungsten wire and then impregnated the polymer matrix with Pd, which was achieved by consecutive dipping of the electrode, first into K_2_PdCl_4_ and then into 0.1 m KCl solution, and final electrochemical treatment to yield metallic Pd.

The authors conducted controlled‐potential electrolysis in carbonate‐containing de‐oxygenated solutions using the prepared electrodes and analyzed the products. To avoid the possibility of obtaining HCO_2_^−^ as the product as a result of degradation of the polymer electrode they also conducted experiments with ^13^C‐enriched carbonate solutions and confirmed the formation of formate using NMR spectroscopy and/or enzyme assays. A faradaic efficiency of 80 % was achieved and the losses were attributed to the formation of H_2_ or formation of palladium hydride through the reaction Pd+*x* H^+^+*x* e^−^ → PdH_*x*_ as well as the reduction of the polymer itself.^102^ The study by Wrighton et al. was one of the earliest showing that heterogeneous catalysis can be achieved for CO_2_ reduction and organic semiconductors can be utilized for electron‐transfer purposes.

**Figure 20:**
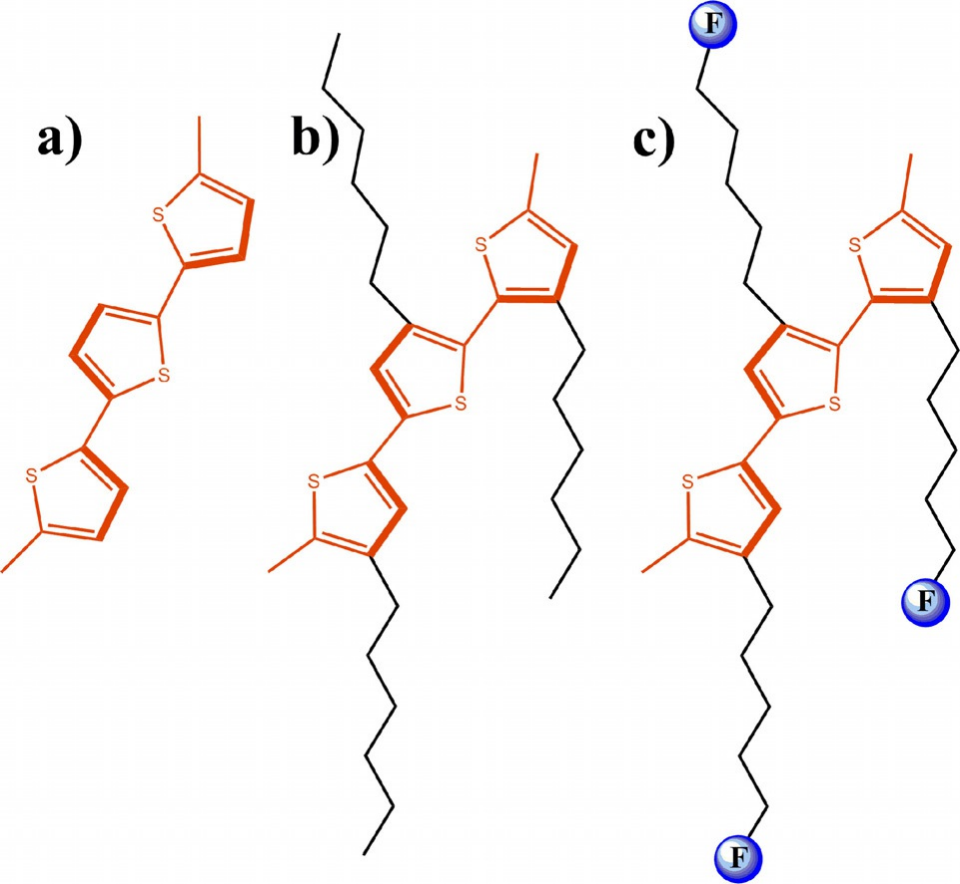
Three generations of conjugated polymers. a) Polymers with good conductivity but low processability; b) polymers with alkyl chains allowing solubility and hence processability; c) polymers with improved and/or new physical/chemical and catalytic properties. Reproduced with permission from Ref. 113.

Another study in which MV^2+^ was used as a functional group attached to a polymer was reported by Sariciftci and co‐workers.^103^ The authors obtained results on methylviologen‐functionalized 3‐alkylpolythiophenes. The study showed characterization of the polymer PTV^2+^ using in situ IR spectroelectrochemistry, UV/Vis and electron spin resonance (ESR). The authors named this new type of polymers as the third generation of conducting polymers (Figure 20) where a solution‐processable and functionalized polymer structure is achieved. ESR studies showed that the electrochemical addressing of viologen moiety is possible. This study did not address its catalytic properties, however, other studies were inspired in which functionalized conducting polymers were used^103^.

**Figure 21:**
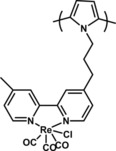
A polypyrrole with [Re(bpy)(CO)_3_Cl] pendant groups.

In terms of polymers that contain Re complexes as the active catalyst in their structure, a study from Meyer and co‐workers^104^ was a door opener. The authors reported the growth of Re‐containing polymers using 4‐vinyl‐4′‐methyl‐2,2′‐bipyridine as both the ligand for Re and the monomer. The authors note that the polymer that formed on the Pt electrode might be result of dimerization or it could be the MeCN‐containing complex as reported previously.^16^ The observed green color of the freshly electropolymerized film suggested the dimerization pathway as the dominant one. However, this color later disappeared. The authors performed controlled potential‐electrolysis in the presence of CO_2_‐saturated solutions at −1550 mV (vs. SCE) to drive electrocatalytic reduction of CO_2_ to CO and reached a faradaic efficiency of 92.3 %. Based on the estimated amount of the catalyst on the polymer it was concluded that the TON was 516. Another important point observed by authors that no formation of CO_3_^2−^ species was observed. This result was in contrast with the results of homogeneous catalysis for which CO and CO_3_^2−^ were detected together. This study was a key success factor for the Re‐containing polymer electrodes in the field and was followed by a study from Cosnier and co‐workers in 1986.^105^ In this work, [Re(bpy)(CO)_3_Cl] was attached to pyrrole at the nitrogen atom and gave a polypyrrole backbone from which the catalyst was a pendant group (Figure 21). If controlled‐potential electrolysis was performed in the presence of CO_2_, the authors found results that contradicted those of their previous study in which they observed formation of CO_3_^2−^ along with oxalate and CO. The faradaic efficiency for CO was 78 % while authors also reported a TON of 236 in this case.^105^ The authors did not explain their choice of polypyrrole as the polymer backbone to drive a reduction reaction although polypyrrole is p‐type in nature; this however might explain the decrease of catalytic activity over time.

Further studies followed investigations of the mechanism behind immobilized Re catalyst in polymer matrices together with different polymers^106^, ^107^ and different metals such as Co, Fe, Ni, Os, and Ru.^108^, ^109^ The use of metal‐free carbon nanofibers for the electrocatalytic reduction of CO_2_ was also reported.^110^, ^111^

**Figure 22:**
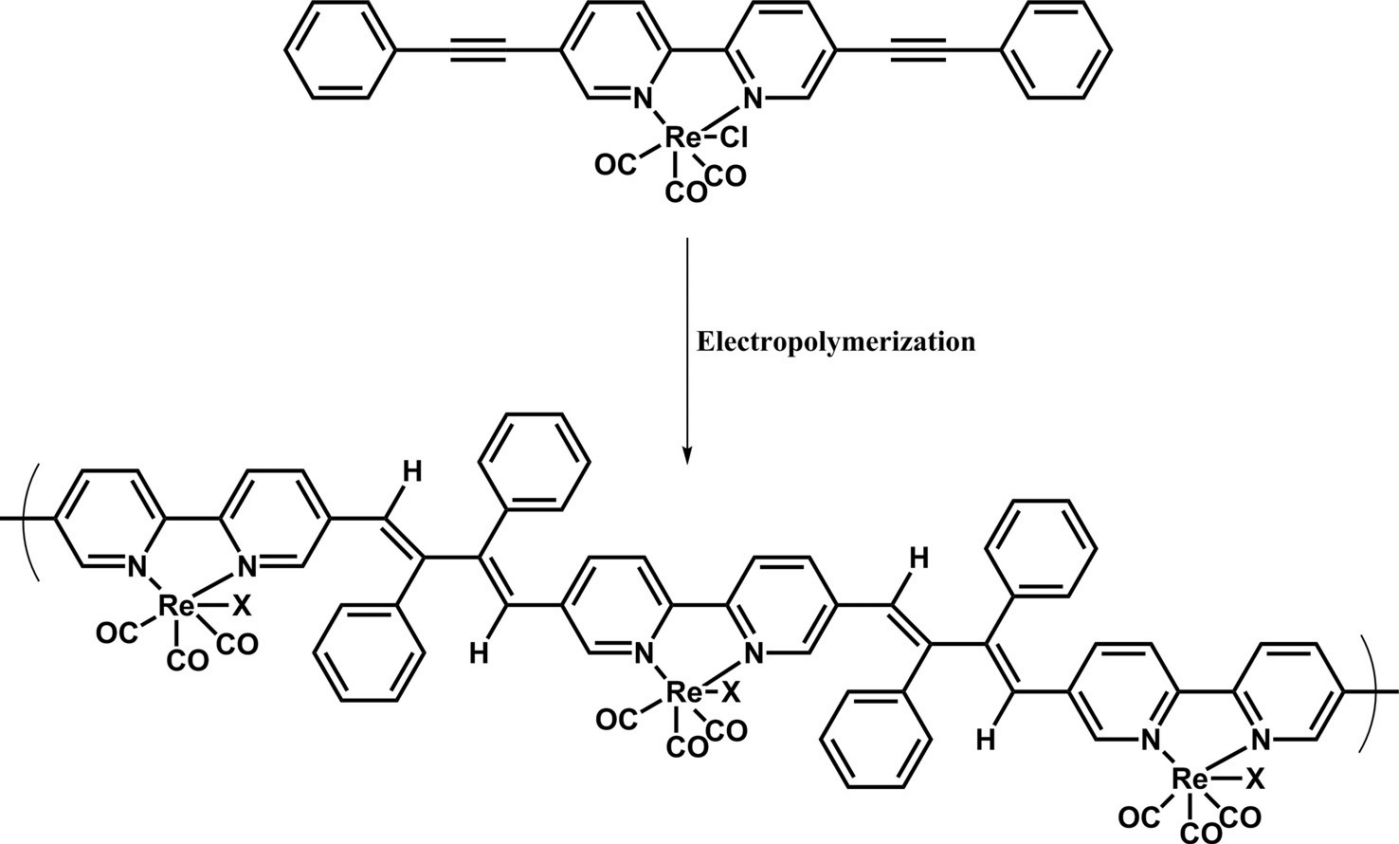
Potentiodynamic formation of Re catalyst film was realized on a Pt electrode in 0.1 m TBAPF_6_ in MeCN with an initial monomer concentration of 2 mm. X represents a chloro ligand or a substituted ligand from the reaction medium.

Another study in which Re complex was incorporated in the main chain of a polymer was reported by Portenkirchner and co‐workers.^112^ The authors used a previously reported modified Re complex and electrochemically polymerized (Figure 22) it onto a Pt electrode potentiodynamically by cycling between −1600 and 200 mV (vs. NHE).

Structural characterization of the film during polymerization was performed using in situ IR spectroelectrochemistry in a flow cell equipped with an Ag/AgCl reference electrode and a Pt plate counter electrode. For this purpose, the film was polymerized onto a ZnSe ATR reflection element. SEM and AFM measurements, as well as spectroscopic characterization, were performed to further investigate the Re‐containing polymer films. Finally, the film was dipped into a solution that was saturated with CO_2_. Cyclic voltammetry revealed a 20‐fold current enhancement in the presence of CO_2_. If held at a constant potential of −1600 mV (vs. NHE) over 60 min, CO was observed as the main product and 33 % faradaic efficiency were achieved. The TON for the Re‐containing polymer film was calculated as 1400.

**Figure 23:**
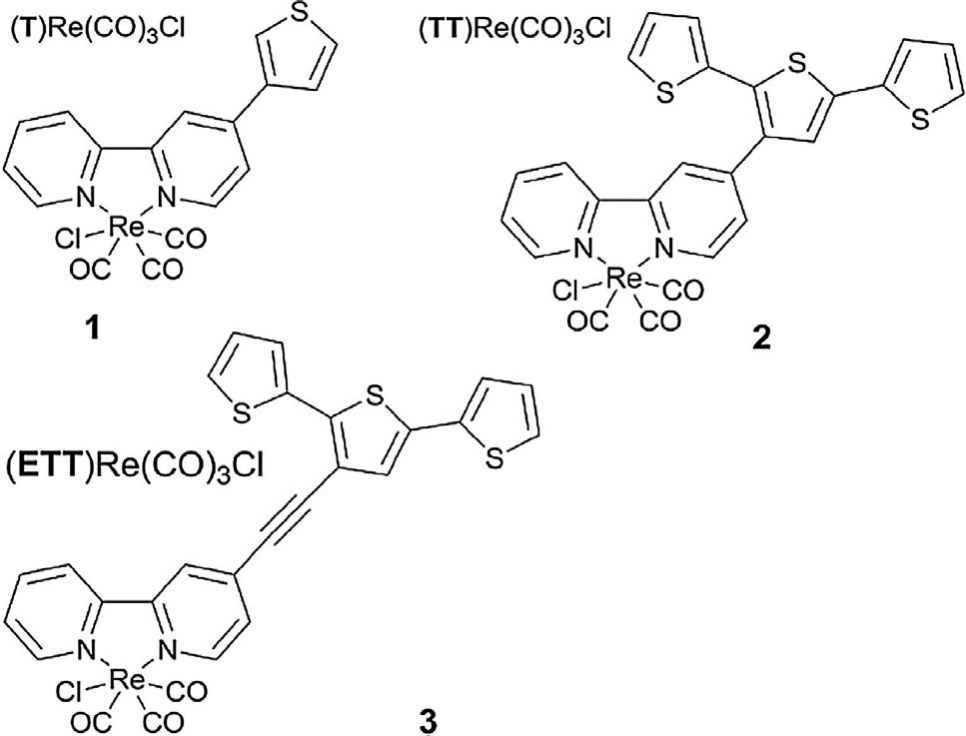
Structures of [Re(bpy)(CO)_3_Cl]‐functionalized thiophene monomers. Reproduced with permission from Ref. 113.

Thiophene, a well‐known and heavily investigated conjugated polymer building block was also used for immobilization of Re complexes. Nervi and co‐workers investigated three thiophene derivatives (Figure 23) with Re pendant groups as conjugated polymer building blocks for electrocatalytic reduction of CO_2_.^113^ The authors investigated the catalytic properties of monomers **2** and **3** as well as their corresponding polymers. The monomers showed a decrease in the catalytic activity after 60 min. However, if the working electrode (glassy carbon) was sonicated for 10 min the catalytic activity was restored. Electrochemically grown polymers were also tested for CO_2_ reduction in CO_2_‐saturated solutions. A faradaic efficiency of 84 % was achieved with a polymer of **2** and 34 % with a polymer of **3** at a potential of −2100 mV (vs. Fc/Fc^+^).^113^

**Figure 24:**
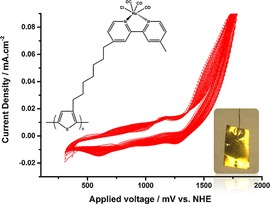
Cyclic voltammogram demonstrating the potentiodynamic polymerization of [3HRe(bpy)(CO)_3_Cl‐Th]. Inset: Photo of a thick film of the polymer. Reproduced with permission from Ref. 114.

**Figure 25:**
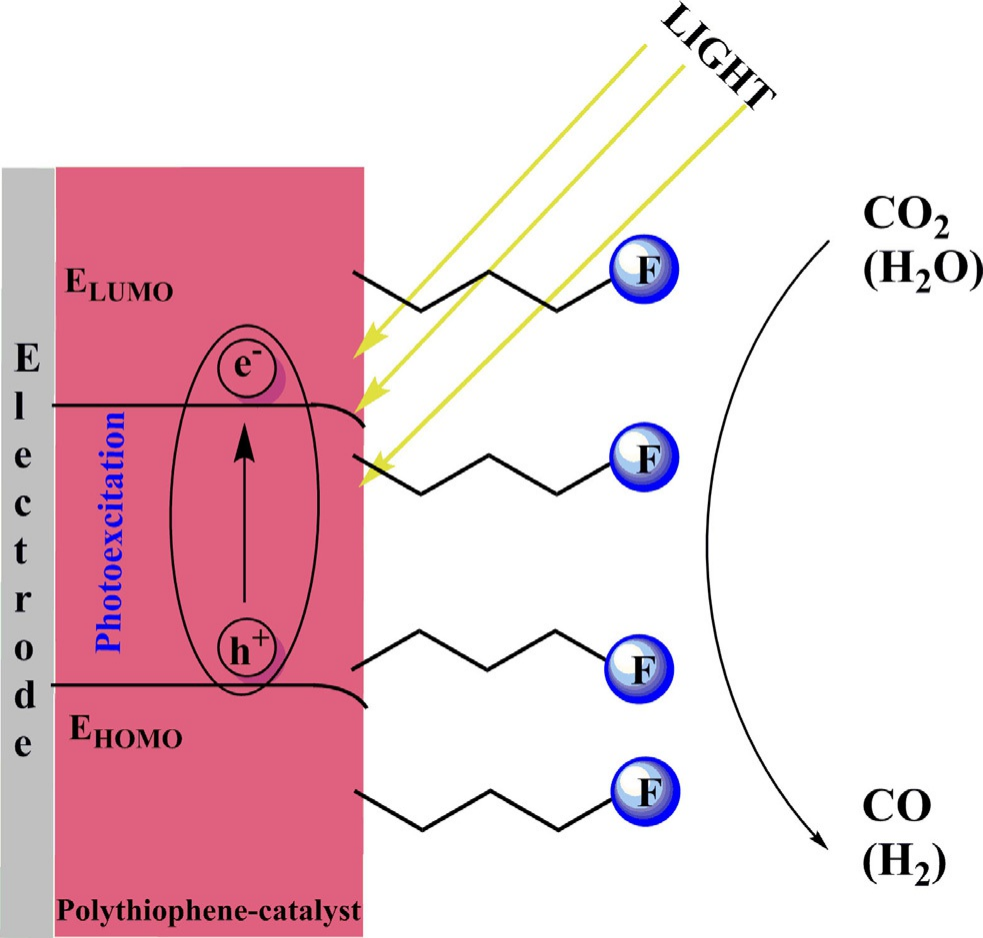
Working principle of the polymeric catalyst upon irradiation with light. H_2_ can be observed as a product if the electrolyte medium is protic. F denotes the catalyst functionalization on the polymer. Reproduced with permission from Ref. 114.

Apaydin et al. investigated polythiophene structures with pendant catalyst groups for the photoelectrochemical reduction of CO_2_.^114^ This is of particular interest because previous studies addressing the use of conjugated polymers lacked the information on how a p‐type material can drive electrons through its backbone unless tunneling was the main driving force. [Re(bpy)(CO)_3_Cl] was attached to thiophene at the 3‐position through an alkyl chain [3HRe(bpy)(CO)_3_Cl‐Th] and then electrochemically polymerized in the presence of boron trifluoride diethyl etherate to yield poly[3HRe(bpy)(CO)_3_Cl‐Th] (Figure 24). After polymerization, the polymer‐modified electrode was dipped into fresh electrolyte solution (0.1 m TBAPF_6_ in MeCN) and scanned either under dark or under illumination in the presence of N_2_. The first and second peaks of the characteristics of Re complex were shifted to more positive potentials by 100 mV when the complex was illuminated, indicating light‐assisted electron transport. The polymer showed a fourfold increase in current upon saturation of the solution with CO_2_ under illuminated conditions. The authors explained the process behind the electron transfer to CO_2_ with the initial formation of an exciton upon illumination followed by electron injection to the valance band of the semiconducting polymer and then by an electron transfer from the polymer to the Re complex to drive CO_2_ reduction (Figure 25). Finally, the authors conducted constant‐potential electrolysis in CO_2_‐saturated solutions at −1500 mV (vs. NHE) to obtain CO as the main product. When 10 % water was added, as suggested previously by Lehn and co‐workers,^14^ CO production dropped significantly and H_2_ evolution prevailed. The authors reached a faradaic efficiency of 2.5 % and a TON of 20 which was calculated from the estimated active sites on the surface.^114^ It is explained in the study that the low faradaic efficiency can be attributed to surface limited reactivity and the choice of a hole extracting electrode. However, this is one of the very few studies where the use of an organic semiconducting polymer and its light absorbing properties to drive photoelectrochemical reduction of CO_2_ is shown.

### 3.4 Immobilized‐Enzyme‐Functionalized Electrodes

The use of enzymes for electrocatalytic reduction of CO_2_ was discussed in section 2.3; however, a mediator (electron shuttle) was always required. Recent studies have focused on the immobilization of enzymes for direct electrochemical addressing by using a conductive platform as well as for protecting them from environmental effects.^115^

**Figure 26:**
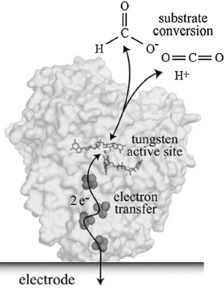
The electrocatalytic interconversion of CO_2_ and formate by a formate dehydrogenase adsorbed onto an electrode surface. Two electrons are transferred from the electrode to the active site (buried inside the insulating protein interior) by iron–sulfur clusters, to reduce CO_2_ to formate, forming a C−H bond. Conversely, if formate is oxidized, the two electrons are transferred from the active site to the electrode. The structure of FDH1 (which contains at least nine iron–sulfur clusters) is not known, so the structure shown is that of the tungsten‐containing formate dehydrogenase from *Desulfovibrio gigas* (PDB ID: 1H0H). Reproduced with permission from Ref. 115.

Hirst and co‐workers used a tungsten‐containing formate dehydrogenase enzyme (FDH1), which was adsorbed on an electrode surface to catalyze CO_2_ reduction to formate (Figure 26).^116^ The authors achieved faradaic efficiencies of around 97–98 % and the enzyme retained its catalytic activity over a pH range of 4–8. Applied potentials to drive the reduction varied between −410 to −810 mV (vs. Ag/AgCl), which are much lower than the potentials required to use organometallic catalysts. FDH1 was the first example of this kind that can catalyze a CO_2_ reduction process with high selectivity and reversibility.^116^

The study from Hirst and co‐workers fueled other heterogeneous electrocatalytic carbon dioxide works in the field. Amao and Shuto reported their findings on the catalytic performance of viologen‐immobilized FDH on an indium tin oxide (ITO) electrode. One end of the viologen was functionalized with a long alkyl chain with a carboxylic acid as the end cap. Carboxylic acid groups served as anchoring groups on a sol–gel‐prepared ITO layer, whereas the other end of the viologen was functionalized with FDH by dipping the electrode into an FDH‐containing solution. If the electrode was biased in a CO_2_‐saturated pyrophosphate buffer solution at −550 mV (vs. Ag/AgCl), it yielded 23 μmol of formic acid after 3 h. The effect of the alkyl chain length was also investigated in the study and the authors reported a trend of increased rate of formic acid production with increasing number of carbon atoms in the alkyl chain, reaching a rate of 7.6 μmol h^−1^ for nine carbon atoms in the chain.

**Figure 27:**
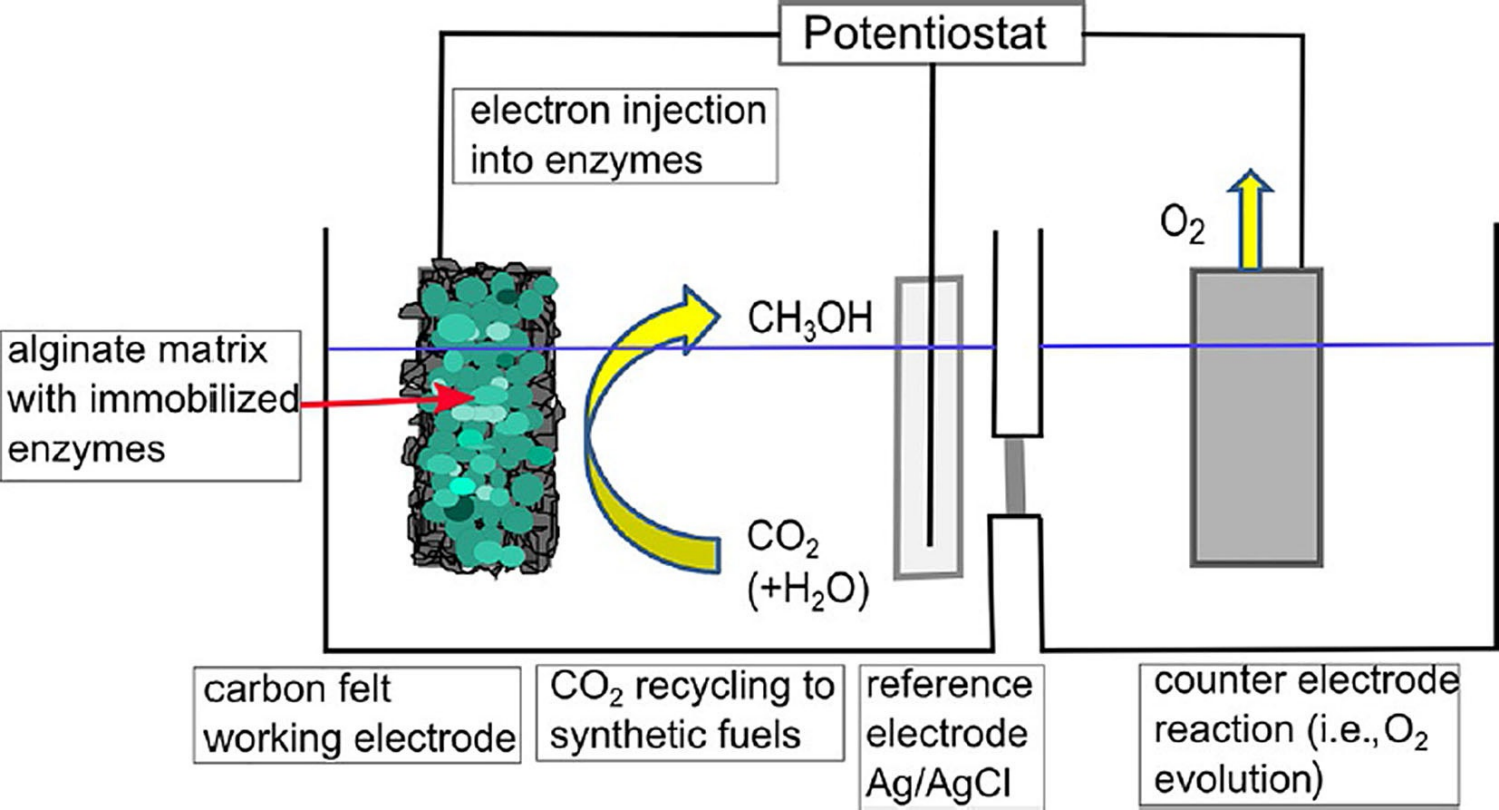
Representation of the electrochemical reduction of CO_2_ using enzymes. Electrons are injected directly into the enzymes, which are immobilized in an alginate–silicate hybrid gel (green) on a carbon felt working electrode. CO_2_ is reduced at the working electrode. Oxidation reactions take place at the counter electrode. Reproduced with permission from Ref. 118.

Schlager et al. demonstrated the immobilization of alcohol dehydrogenase on highly porous carbon felt electrodes using a alginate–silicate hybrid gel as the immobilization matrix.^117^ Alcohol dehydrogenase catalyzed the reduction of butyraldehyde to butanol at a potential of −600 mV (vs. Ag/AgCl) with a faradaic efficiency of 40 %. The authors also performed a control experiment in which they immobilized the enzyme in the same matrix together with NADH as an electron donor to check the activity of the enzymes over time. They achieved a 96 % conversion if NADH was the electron donor.^117^ Formation of methanol using the same method with a faradaic efficiency of 10 % was also demonstrated at a constant potential of −1200 mV (vs. Ag/AgCl)^118^ (Figure 27).

**Scheme 8:**
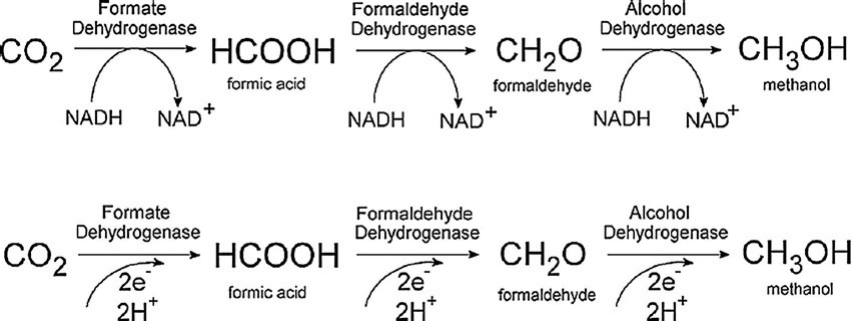
Mechanisms for CO_2_ reduction catalyzed by dehydrogenases. Three‐step reduction of CO_2_ to methanol using NADH as a sacrificial coenzyme (top) and through a direct electron transfer to the enzyme without coenzyme (bottom). Reproduced with permission from Ref. 118.

By expanding the idea of biocatalytic electrochemistry to include multiple enzymes immobilized on an electrode, Schlager et al. showed that CO_2_ can be reduced to methanol in a triple cascade.^118^ This study showed the successful immobilization and electrochemical utilization of three enzymes to achieve bioelectrocatalytic reduction of CO_2_. NADH can be replaced in the reaction cascade with direct electron injection to the enzymes (Scheme 8).^118^ Biocatalytic systems such as enzymes and bacteria work in the mild conditions of room temperature and atmospheric pressure, and are superior to all other catalysts in terms of selectivity.

## 4 Summary and Outlook

We reviewed previous work in the field of CO_2_ reduction that used organometallic, organic and bioorganic catalysts to initiate electrocatalytic, photoelectrocatalytic and bioelectrocatalytic reactions.

Reduction of CO_2_ using a heterogeneous catalytic approach has the following advantages: immobilization of the catalyst material on the working electrode can allow the direct use of the catalyst, bypassing the diffusion step. Another advantage of course is the reusability of catalyst material without the need to recover it from the mixed chemical medium of the products and catalyst together. The economics of electrocatalytic CO_2_ reduction is still under discussion as to whether it will be feasible or not in the near future. Recent work from Kenis et al.^119^ demonstrated the use of a gross margin model for calculating the economics of CO_2_ reduction. The gross margin model is defined as the difference between the revenue and the cost of goods, divided by the revenue. This shows that CO and HCOOH are economically the most feasible products to pursue in terms of required potential and faradaic efficiency. However, improvements in catalyst durability and energy efficiency are still needed. However, the activation of CO_2_ can be energy demanding and we should certainly make use of renewable energies. Organic p‐type semiconductors can be used as electron‐transfer media to provide photogenerated electrons to catalyst materials that are immobilized on the surfaces of semiconductors. Hybrid systems in which the catalyst is of biological origin and the electron source is ideally a photoelectroactive compound can pave the way toward energy efficient and selective conversion of CO_2_. Biocatalytic systems work at room temperature and atmospheric pressure and have superior selectivity. These can be major factors in calculating the economics of large‐scale CO_2_ reduction processes.

The cyclic use of carbon in the ways described in this Review will create a carbon‐neutral energy vector, which is important for transforming our energy‐producing sectors.

## Conflict of interest

*The authors declare no conflict of interest*.

## Biographical Information

Dogukan H. Apaydin obtained his BSc degree in chemistry (2010) from Istanbul Technical University and his MSc degree in polymer science and technology (2012) from Middle East Technical University in Turkey. He joined the Linz Institute for Organic Solar Cells (LIOS) in November 2012 and is currently a PhD student focusing on electrochemically assisted carbon capture and (photo)electrochemical reduction of carbon dioxide using organic semiconductor electrodes.

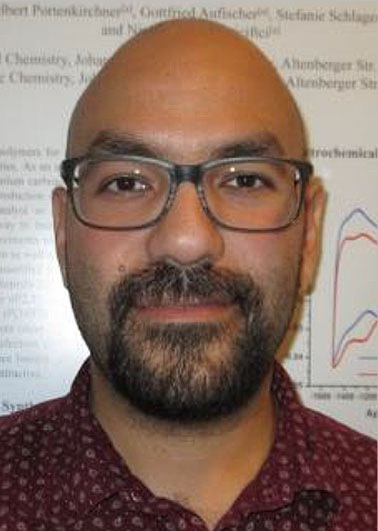

## Biographical Information

Stefanie Schlager joined the LIOS group in 2010 and completed her studies in technical chemistry and chemical engineering at the Johannes Kepler University Linz in 2011. The topic of her first diploma thesis was organic Schottky diodes, which was followed by her second diploma thesis and PhD at LIOS, where she focused on semiconductor–electrolyte interfaces for the application of electrochemical, biocatalytic, and bioelectrocatalytic reduction of CO_2_. From the beginning of 2016, she was a postdoctoral assistant at LIOS and her scientific work was based on the further development of microbial electrosynthetic and electroenzymatic conversion processes. Since the beginning of 2017, she has worked as a project manager for Lenzing AG.

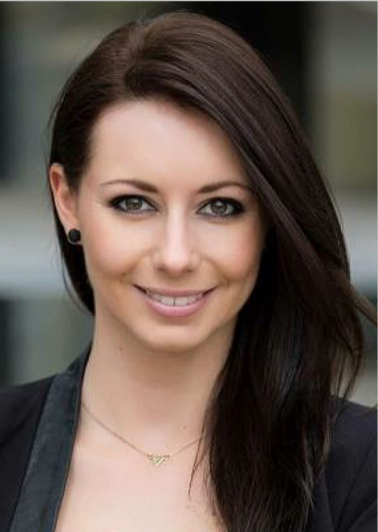

## Biographical Information

Engelbert Portenkirchner is a postdoctoral researcher at the Institute for Physical Chemistry at the University of Innsbruck. In 2009 he started his PhD studies at the Linz Institute for Organic Solar Cells (LIOS) at the Johannes Kepler University Linz, where he graduated with a Dr. techn. degree in 2014. His present research interests are in the field of electro‐ and photocatalytic CO_2_ reduction, as well as materials synthesis and characterization for energy conversion and storage with application in (post) lithium‐ion batteries. He is the co‐author of about 20 publications in international, peer‐reviewed journals.

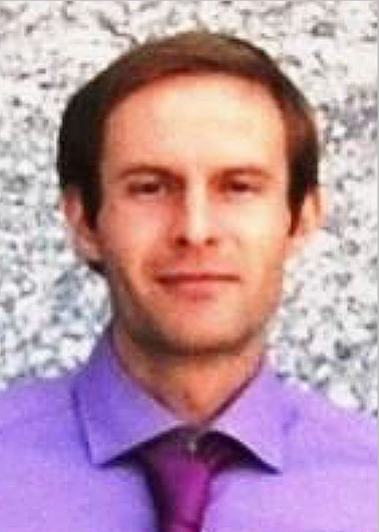

## Biographical Information

Professor Niyazi Serdar Sariciftci is Ordinarius Professor of Physical Chemistry and the Founding Director of the Linz Institute for Organic Solar Cells (LIOS) at the Johannes Kepler University Linz (Austria). He studied at the University of Vienna (Austria) and graduated with a PhD in physics in 1989. After two years of postdoctoral work at the University of Stuttgart (Germany), he joined the Institute for Polymers and Organic Solids at the University of California, Santa Barbara (USA), with Professor Alan J. Heeger, a 2000 Nobel Laureate in Chemistry. His major contributions are in the fields of photoinduced optical, magnetic resonance and transport phenomena in semiconducting and metallic polymers. He is the inventor of conjugated polymer‐ and fullerene‐based bulk heterojunction solar cells. Professor Sariciftci has published over 600 articles and, with over 50 000 citations, he is one of the most cited scientists in material science (2011, Thomson Reuters ranking No. 14 of the world best material scientists). As of January 2017, Professor Sariciftci has *h*‐indices of 86 (ISI Thompson) and over 103 (Google Scholar). Professor Sariciftci has composed eight books and educated several academic and industrial scientists. He has also initiated seven spin‐off companies involved in organic optoelectronics. He has been the recipient of several prizes, among them the National Science Prize of Turkey 2006 and the Austrian Scientists of the Year Prize for Research 2008. He received the Medal for Humanity of the City of Linz 2009 and the Kardinal Prize for Science of the Archbishop in Vienna 2010. In 2012, he was awarded the prestigious Wittgenstein Prize of Austria. He is a Fellow of the Royal Society of Chemistry (FRSC), a Fellow of SPIE, and a member of several societies, including the American Chemical Society, the Materials Research Society, the Austrian Chemical Society, and the Austrian Physical Society. He was selected as a corresponding member of the Academy of Science in Austria (ÖAW). Professor Sariciftci has been awarded honorary doctorates by the Abo Academy in Finland in 2011 and the University of Bucharest in Romania in 2012. Recently, Professor Sariciftci received the TÜBA Science Prize from the Turkish Academy of Sciences (2015).